# Comparative efficiency and safety of potassium competitive acid blockers *versus* Lansoprazole in peptic ulcer: a systematic review and meta-analysis

**DOI:** 10.3389/fphar.2023.1304552

**Published:** 2024-01-11

**Authors:** Yongqi Dong, Hongyan Xu, Zhihuan Zhang, Zhihang Zhou, Qiang Zhang

**Affiliations:** ^1^ Department of Gastroenterology, Wushan County People’s Hospital of Chongqing, Chongqing, China; ^2^ Department of Infectious Disease, The Second Affiliated Hospital of Chongqing Medical University, Chongqing, China; ^3^ Department of Rheumatology and Immunology, The Second Affiliated Hospital of Chongqing Medical University, Chongqing, China; ^4^ Department of Gastroenterology, The Second Affiliated Hospital of Chongqing Medical University, Chongqing, China; ^5^ Department of Spinal Surgery, Wushan County People’s Hospital of Chongqing, Chongqing, China

**Keywords:** potassium competitive acid blocker, lansoprazole, peptic ulcer, vonoprazan, tegoprazan, keverprazan

## Abstract

**Background:** Lansoprazole, a proton-pump inhibitor (PPI), is the primary therapy for peptic ulcers (PU). Potassium competitive acid blockers (P-CAB) offer an alternative for acid suppression. However, the efficacy and safety of P-CABs *versus* lansoprazole in the management of PU has not been evaluated.

**Methods:** Five databases were searched for randomized clinical trials in English until 31 August 2023. Data extraction provided outcome counts for ulcer healing, recurrent NSAID-related ulcer, and adverse events. The pooled effect, presented as rate difference (RD), was stratified by ulcer location, follow-up time, and the types of P-CAB, along with their corresponding 95% confidence intervals (95% CI).

**Results:** The pooled healing rates of peptic ulcers were 95.3% (1,100/1,154) and 95.0% (945/995) for P-CABs and lansoprazole, respectively (RD: 0.4%, 95% CI: −1.4%–2.3%). The lower bounds of the 95% CI fell within the predefined non-inferiority margin of −6%. In subgroup analyses base on ulcer location, and follow-up time also demonstrated non-inferiority. The drug-related treatment-emergent adverse events (TEAEs) did not differ significantly among groups (RR: 0.997, 95% CI: 0.949–1.046, *p* = 0.893). However, P-CAB treatment was associated with an increased risk of the serious adverse events compared to lansoprazole (RR: 1.325, 95% CI: 1.005–1.747, *p* = 0.046).

**Conclusion:** P-CABs demonstrated non-inferiority to lansoprazole in the management of peptic ulcer. The safety and tolerability profile are comparable, with similar TEAEs rates. However, P-CABs appear to have a higher risk of serious adverse events.

**Systematic Review Registration:** https://www.crd.york.ac.uk/PROSPERO/display_record.php?RecordID=458361 Identifier: PROSPERO (No. CRD42023458361).

## Introduction

Peptic ulcer (PU) is a prevalent disease with a lifetime prevalence ranging from 5% to 10% in the general population and an annual incidence rate of 0.1%–0.3%. PU usually occur in either the stomach [gastric ulcers (GU)] or the duodenum [duodenal ulcers (DU)] (Lanas and Chan, 2017). The primary etiological factors associated with PU are *H. pylori (H. pylori)* infection and the utilization of NSAIDs (Sung, Kuipers, and El-Serag, 2009). Although with the utilization of potent anti-secretory medications and the widespread adoption of *H. pylori* eradication therapy, both the incidence and mortality rates associated with PU have gradually decreased. Management of PU remains challenging at present due to increased antimicrobial resistance and the widespread use of antithrombotic therapy in the elderly population (Nagasue et al., 2015).

Proton pump inhibitors (PPI) are widely used for PU treatment, and their therapeutic effects are generally considered satisfactory. However, there are certain limitations that need to be addressed (Strand, Kim, and Peura, 2017). Potassium-competitive acid blockers (P-CAB) represent a novel class of medications known for their rapid and effective anti-secretory activity. Unlike traditional PPIs, P-CABs can promptly inhibit proton pumps without the need for gastric acid activation and do not require food intake. So, they provide a fast onset of action, delivering their full effect from the very first dose. P-CABs also have a longer plasma half-life compared to PPIs, making them effective in inhibiting nocturnal gastric acid (Sakurai et al., 2015; Mori and Suzuki, 2019). Additionally, the blood drug concentration of P-CABs was not significantly influenced by CYP2C19 gene polymorphism, it makes efficacy monitoring more convenient (Kagami et al., 2016).

Previous meta-analyses have examined the non-inferiority of P-CABs compared with lansoprazole in the treatment of *H. pylori* infection (Jung, Kim, and Park, 2017), artificial ulcers after endoscopic submucosal dissection (ESD) (Liu et al., 2019) and esophagitis (including reflux and erosive esophagitis) (Miwa et al., 2019; Yang et al., 2022). Based on clinical and evidence-based medicine outcomes, P-CABs are currently recommended in guidelines for the management of two acid-related diseases: esophagitis and *H. pylori* eradication therapy (Jung et al., 2021; Iwakiri et al., 2022; Zhou et al., 2022). However, there is currently a lack of comprehensive summaries regarding the use of P-CABs for PU. Therefore, we conducted this meta-analysis to consolidate contemporary clinical evidence and evaluate the therapeutic the non-inferiority efficacy and safety of P-CABs compared with lansoprazole for the management of PU, encompassing both the treatment of PU and the prevention of NSAID-related ulcer recurrence.

## Methods

This systematic review and meta-analysis meticulously followed the guidelines outlined in the Preferred Reporting Items for Systematic Reviews and Meta-Analyses (PRISMA) statement (Page et al., 2021). Our study has been registered with PROSPERO under the registration number CRD42023458361. The complete dataset is accessible online.

### Search strategy

We conducted a comprehensive and systematic search across five databases (Cochrane, Embase, PubMed, Web of Science, and Scopus) to identify double-blind randomized controlled trials (RCTs). The search period included studies from their inception up to 31 August 2023. Additionally, we manually reviewed the reference lists of relevant studies for potentially related research. Our search terms encompassed keywords such as “peptic ulcer,” “gastric ulcer,” “duodenal ulcer,” “P-CAB,” “vonoprazan,” “PPI,” “lansoprazole, " and “potassium competitive acid blocker,” among others. A detailed search strategy is presented in Appendix 2. We limited our search to publications written in English. Two independent reviewers (Yongqi Dong and Hongyan Xu) independently assessed the titles and abstracts. For studies meeting the inclusion criteria, we conducted a thorough review of the full text. Any discrepancies between reviewers were resolved through consensus or referred to a third reviewer (Zhihuan Zhang).

### Selection criteria

This study adheres to the “PICOS” framework with the following criteria: 1) Patients: Individuals with PU requiring treatment or those with long-term NSAID therapy necessitating prevention of NSAID-related ulcer recurrence. 2) Intervention: P-CABs. 3) Control: Lansoprazole. 4) Outcomes: Primary Outcome: Proportion of patients with endoscopically confirmed healing at Week 6 (for DU) or Week 8 (for GU). Secondary Outcomes: i) Recurrence rate of peptic ulcers at 12 and 24 weeks in patients with long-term NSAID therapy. ii) Incidence rate of treatment-emergent adverse events (TEAE) in both the P-CABs and lansoprazole groups. 5) Study Designs Chosen: RCTs.

The inclusion criteria were as follows: 1) Patients aged over 18 years with the primary objective of ulcer treatment or prevention of NSAID-related ulcer. 2) prospective phase II or phase III randomized clinical trials. 3) The control group received lansoprazole at dosages recommended by guidelines (Matheson and Jarvis, 2001). 4) All patients had confirmed active ulcers of A1 or A2 stage based on the Sakita-Miwa classification (Sakita et al., 1971) or the presence of ulcer scars through endoscopy within 14 days prior to enrollment. 5) Detailed reporting of the occurrence of events and adverse events in both the treatment and control groups.

Studies meeting any of the following exclusion criteria were excluded: 1) Guidelines, expert position or consensus, brief reports, case reports, letters, comments, or protocol studies; review and meta-analysis articles; 2) Cohort, case-control, pharmacokinetic, animal or cell studies. 3) Different outcomes of interest. 4) Studies with incomplete or duplicate data.

### Data extraction

Two independent reviewers (Yongqi Dong and Hongyan Xu) conducted the data extraction process. Data extraction from the relevant studies included following information: first author, year of publication, treatment dosages, treatment duration, follow-up time, non-inferiority margin, and the total number of patients receiving treatment. Primary outcomes required the precise count of events, while secondary outcomes were considered only if they were reported in three or more studies. Any discrepancies between the two reviewers were resolved by a third reviewer (Qiang Zhang).

### Methodological quality

The Cochrane Risk-of-Bias Tool for Randomized Trials (RoB 2) (Cumpston et al., 2022) was used to assess the risk of bias by Review Manager software (Version 5.3). The Cochrane bias risk criteria included the following six components: selection bias, performance bias, detection bias, attrition bias, reporting bias, and other sources of bias.

### Publication bias

When the number of included studies >10, the small-sample study effect was evaluated both qualitatively and quantitatively (Cumpston et al., 2019). Qualitative assessment involved the use of a funnel plot, while quantitative assessment was performed using Egger’s test.

### Subgroup and sensitivity analyses

Subgroup analyses have been conducted based on ulcer location, follow-up time, types of P-CAB, and serious adverse events. Sensitivity analyses have been performed, which include leave-one-out analysis, Galbraith plots, L’Abbé plots, and the manual removal of studies with high heterogeneity to assess the robustness of the results.

### Statistical analysis

We conducted a meta-analysis using STATA 16.0 to summarize the study findings. Heterogeneity among the included studies was assessed using Cochrane χ^2^ and I^2^ statistics. In cases of significant heterogeneity (I^2^ > 50%), we used the random-effects model to summarize the effect size; otherwise, the fixed-effect model was used. Analyses were conducted as full analysis set (FAS) instead of per protocol (PP) analyses to reduces the risk of type I error, as the former approach is more conservative (Bai et al., 2021). The Mantel-Haenszel method was used to calculate the pooled risk ratio (RR) and its corresponding 95% confidence interval (95% CI) for ulcer healing rates and TEAEs.

As all the studies included in our review were non-inferiority trials, and none of them could prove the superiority of PCABs over lansoprazole in the treatment of ulcers. Moreover, RR is primarily applied in studies designed for superiority analysis and may not suitable for non-inferiority trials (Food and Administration, 2016). Consequently, we extracted non-inferiority margins from all the included studies and opted for the most conservative margin to ensure the interpretability of non-inferiority. The validity of non-inferiority when the lower 95% CI of the pooled rate difference (RD) fell within these non-inferiority margins (Treadwell et al., 2012). Statistical significance was considered when a two-tailed *p*-value is less than 0.05.

## Results

### Study inclusions

**FIGURE 1:**
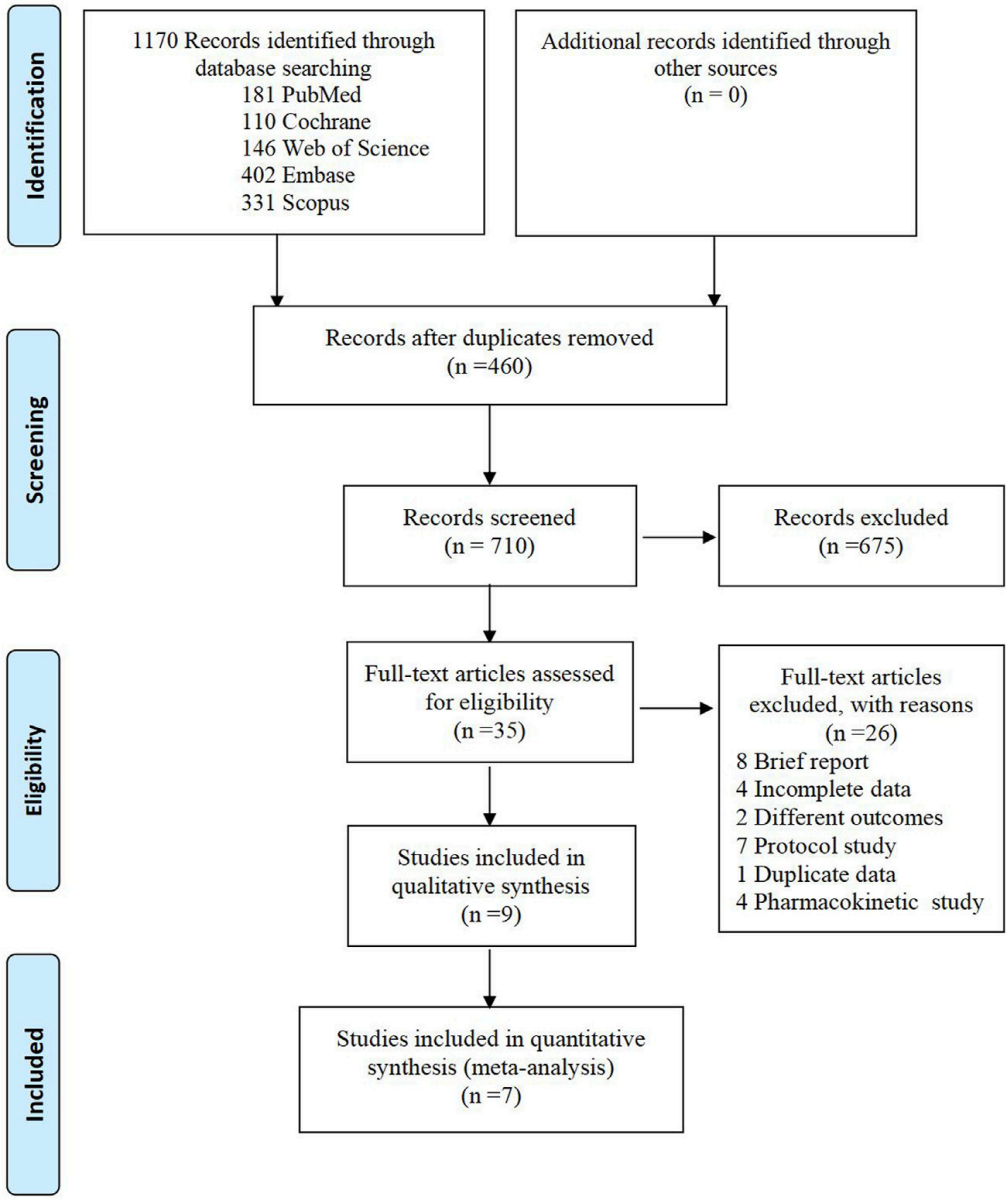
Flow chart of study selection.

A total of 1,170 articles were initially identified through the search process. After removing 460 duplicates, we carefully reviewed the titles and abstracts of the remaining 710 studies. Subsequently, we assessed the full text of 35 studies for eligibility. 26 studies were excluded for the following reasons: 8 were brief reports, 4 had incomplete data, 2 examined different outcomes of interest, 4 were pharmacokinetic studies, 7 were protocol studies, and 1 was duplicate data. Finally, seven randomized controlled trials (RCTs) were included in the meta-analysis. The detailed search process is illustrated in Figure 1.

### Characteristics and quality assessment of the studies

Table 1 summarized the characteristics of the included studies. Our meta-analysis incorporated five RCTs for PU treatment, two RCTs for NSAID-related ulcer prevention, and all seven studies for safety analysis. In total, 2,149 individuals participated, with 1,154 in the P-CAB group and 995 in the lansoprazole group. The P-CABs included vonoprazan, tegoprazan, and keverprazan. Lansoprazole was administered at a standard dose of 15 mg once daily for NSAID-related ulcer prevention and 30 mg once daily for PU treatment. Of these studies, three were conducted in Japan, one in Korea, and three in China. The treatment duration was typically 6 weeks for DU and 8 weeks for GU in five studies, while the other two studies focused on NSAID-related ulcer prevention over 24 weeks. Non-inferiority margins ranged from 6% to 8% for DU, 8%–8.54% for GU, and 8.3%–8.7% for NSAID-related ulcer.

**TABLE 1:** Feature of included studies.

Nationality	Treatment	Control	Treatment duration	Outcome	Outcome measure	Non-inferiority margin
Japan	Vonoprazan 20 mg once daily	Lansoprazole 30 mg once daily	6 weeks for GU	DU	GU: RD: 0.3% (95% CI: 4.750–4.208)	6% for DU
				GU	DU: RD: −2.8% (95% CI: −6.400–0.745)	
			8 weeks for DU	TEAE	TEAE: 65% VS 79% in GU	8% for GU
					63% VS 53% in DU	
Japan	Vonoprazan 10 mg once daily	Lansoprazole 15 mg once daily	24 weeks	recurrence of PU	10 mg: PD: –2.2% (95% CI: –6.2%−1.8%)	8.3%
					20 mg: PD: –2.1% (95% CI: –6.1%−2.0%)	
	Vonoprazan 20 mg once daily			TEAE	TEAE	
					84.4% (10 mg) VS 82.5% (20 mg) VS 88.1%	
Japan	Vonoprazan 10 mg once daily	Lansoprazole 15 mg once daily	24 weeks	recurrence of PU	10 mg: PD: −2.3% (95% CI: −4.743–0.124)	8.7%
					20 mg: PD: −1.3% (95% CI: −4.095–1.523)	
	Vonoprazan 20 mg once daily			TEAE	TEAE	
					87.6% (10 mg) VS 87.1% (20 mg) VS 84.8%	
Korea	Tegoprazan 50 mg once daily	Lansoprazole 30 mg once daily	4 weeks	GU	50 mg: RD: −0.91% (95% CI: −7.98–6.09)	8.54%
					100 mg: RD: −0.75% (95% CI: −7.69–6.31)	
	Tegoprazan 100 mg once daily		8 Weeks (if not healed)	TEAE	TEAE	
					17.65% (50 mg) VS 22.55% (100 mg) VS 25.00%	
China	Keverprazan 20 mg once daily	Lansoprazole 30 mg once daily	6 weeks	DU	healing rate	-
					4 weeks: 87.27% (20 mg) VS 90.16% (30 mg) VS 79.69%	
	Keverprazan 30 mg once daily			TEAE	6 weeks: 96.36% (20 mg) VS 98.36% (30 mg) VS 92.19%	
					TEAE	
					43.64% (20 mg) VS 63.93% (30 mg) VS 64.06%	
China	Vonoprazan 20 mg once daily	Lansoprazole 30 mg once daily	6 weeks	DU	RD: 0.4% (95%CI: −3.00–3.79)	6%
				TEAE	TEAE: 74.1% VS 64.9%	
China	Keverprazan 20 mg once daily	Lansoprazole 30 mg once daily	6 weeks	DU	RD: 1.2% (95% CI: 24.0%–6.5%)	8%
				TEAE	TEAE: 57.8% VS 59.0%	

CI:confidence interval; DU: duodenal ulcer; GU: gastric ulcer; PD: percentage difference; PU: peptic ulcer; RD: rate difference; TEAE: treatment-emergent adverse event.

**FIGURE 2:**
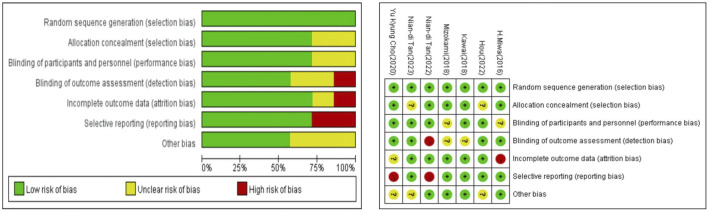
Summary of risk of bias assessment. Risk of bias of included randomized controlled trials or prospective trials (review authors’ judgments about each risk of bias item for each included study: +, low risk; −, high risk; ?, unclear risk).

The overall risk of bias is depicted in Figure 2, with low risk observed in all trials except for potential bias due to missing data that could impact outcomes, and a small proportion of predefined outcomes that could not be obtained.

### Efficacy analysis

**FIGURE 3:**
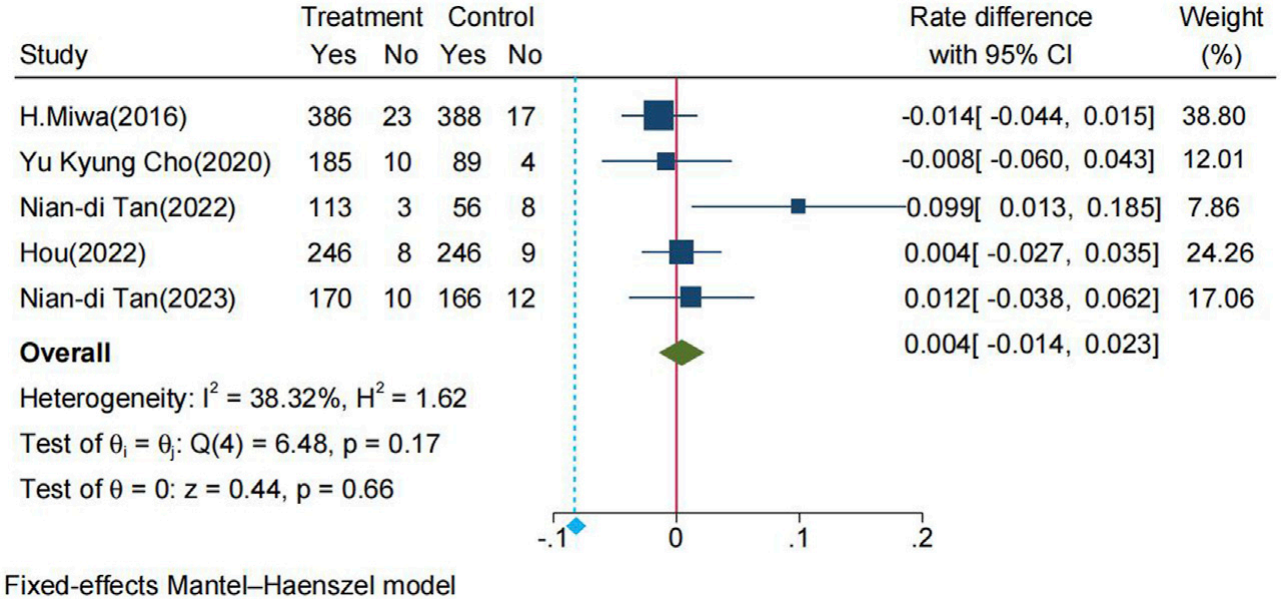
Forrest plot for healing rate of PU between the P-CABs and lansoprazole groups. (fixed-effects model); CI: confidence interval.

The pooled healing rates of PU in the P-CAB and lansoprazole groups were 95.3% and 95.0%, respectively. The RD between P-CAB and lansoprazole in the FAS analysis was 0.4% (95% CI: −1.4%−2.3%). The lower 95% CI of the pooled RD (−1.4%) fell within the predefined non-inferiority margin of −6%, thereby the non-inferiority of P-CABs compared to lansoprazole was confirmed (Figure 3). The pooled RR for healing rate was 1.00 (95% CI: 0.98–1.02, *p* = 0.66), indicating that P-CABs is not superior to lansoprazole in the treatment of PU (Supplementary Figure S6.1).

**FIGURE 4:**
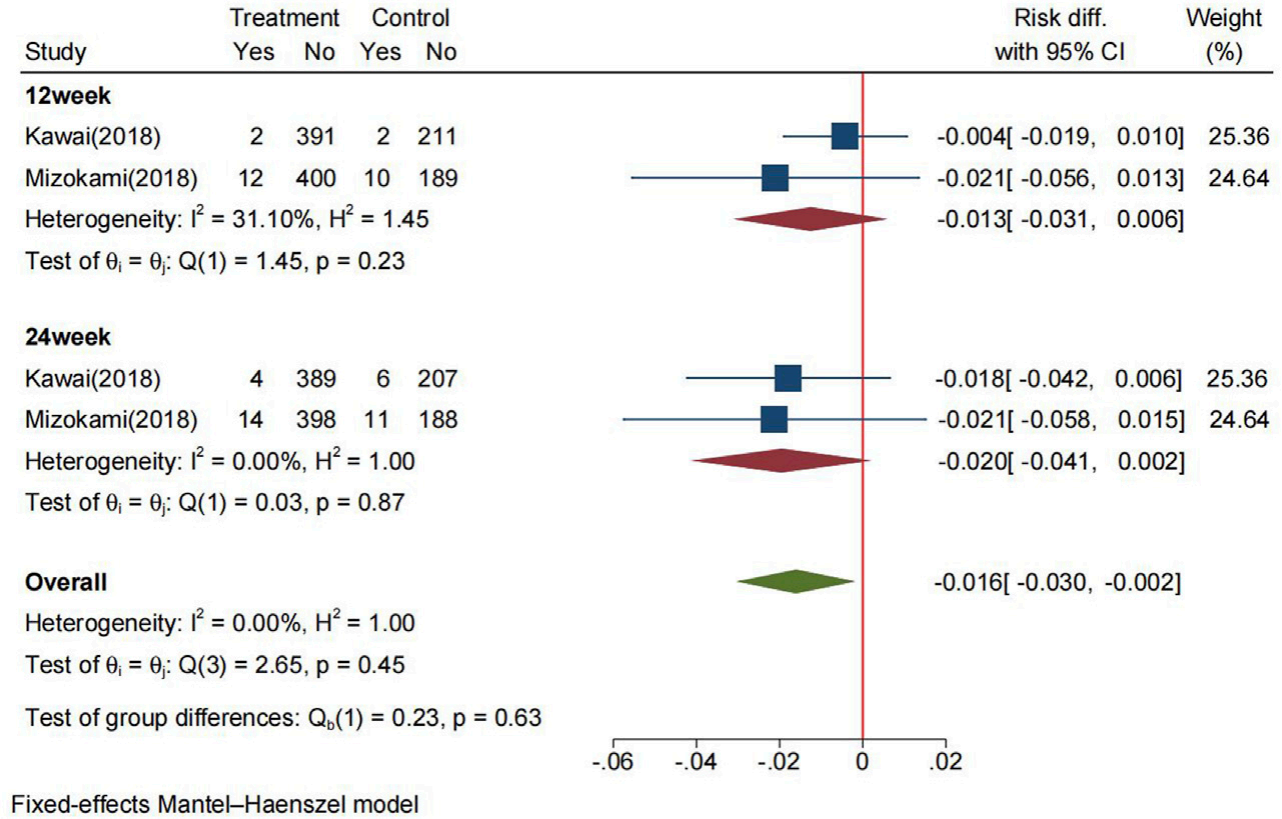
Forrest plot for the recurrence of NSAID-related ulcer between the P-CABs and lansoprazole groups (fix-effects model) CI: confidence interval.

The pooled recurrence rate of PU at 24 weeks in the P-CABs and lansoprazole groups were 3.4% and 5.5%, respectively. The RD between the two groups in the FAS analysis was −2.0% (95% CI: −4.1%−0.2%), the RD at 12 weeks was −1.3% (95% CI: −3.1%−0.6%) (Figure 4). The pooled RR for recurrence rate was 0.53 (95% CI: 0.27–1.01, *p* = 0.48) (Supplementary Figure S6.2). The above results indicate that P-CABs is not superior but no-inferior to lansoprazole in preventing the recurrence of NSAID-related ulcer.

### Safety analysis

**FIGURE 5:**
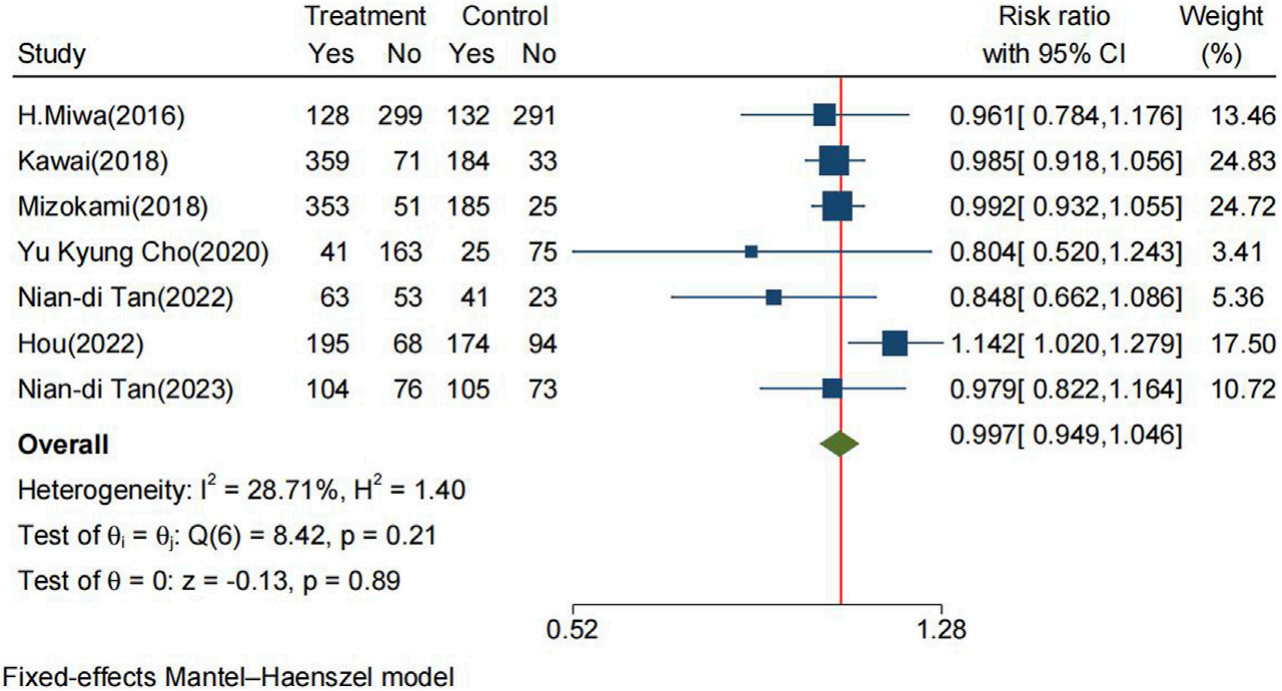
Forrest plot for TEAEs between the P-CABs and lansoprazole groups. (fix-effects model) CI: confidence interval, TEAEs: treatment-emergent adverse events.

The incidence rate of TEAEs in the P-CABs and lansoprazole groups within the FAS was 61.4% and 57.9%, respectively. The RR for TEAEs was 0.997 (95% CI: 0.949–1.046, *p* = 0.89) (Figure 5). However, P-CABs treatment was associated with an elevated risk of serious adverse events compared to lansoprazole (RR: 1.325, 95% CI: 1.005–1.747, *p* = 0.046) (Supplementary Figure S5.4).

### Subgroup analysis

In subgroup analyses base on ulcer location, the RD was 0.8% (95% CI: −2.8%–4.4%) in the GU group and −0.5% (95% CI: −3.9%–2.9%) in the DU group, all the lower bounds of the 95% CI fell within the non-inferiority margin of −6% (Supplementary Figure S5.1). There was no significant of RR in both DU and GU group (DU: RR: 1.01, 95% CI: 0.97–1.05, *p* = 0.06; GU: RR: 0.99, 95% CI: 0.96–1.03, *p* = 0.63) (Supplementary Figure S6.3).

In subgroup analyses base on follow-up time (DU: 4 weeks: RD: 1.3%, 95% CI: −1.9%–4.4%; 6 weeks: RD: 0.8%, 95% CI: −2.8%–4.4%; GU: 4 weeks: RD: 0.8%, 95% CI: −6.6%–5.0%; 8 weeks: RD: −0.5%, 95% CI: −3.9%–2.9%). The lower 95% CI of the RD at 4 weeks in GU was −6.6%, which falls outside the predefined non-inferiority margin of −6%. This indicated that P-CABs is not non-inferior to lansoprazole in the 4-week healing effect of GU (Supplementary Figure S5.2). The pooled RR was insignificant between two groups (DU: 4 weeks: RR: 1.01, 95% CI:0.98–1.05, *p* = 0.43; 6 weeks: RR: 1.01, 95% CI: 0.97–1.05, *p* = 0.06; GU: 4 weeks: RR: 0.99, 95% CI: 0.92–1.06, *p* = 0.38; 8 weeks: RR:1.00, 95% CI: 0.96–1.03, *p* = 0.86) (Supplementary Figure S6.4).

In subgroup analyses base on the types of P-CAB, including vonoprazan, tegoprazan, and keverprazan, all were non-inferior to lansoprazole in the treatment of PU. The RD compared to lansoprazole was −0.6% (95% CI: −2.7%–1.6%), −0.8% (95% CI: −6%–4.3%), and 4.8% (95% CI: −3.6%–13.2%), respectively (Supplementary Figure S5.3). The RR of these three P-CABs compared to lansoprazole was insignificant (vonoprazan: RR: 0.99, 95% CI: 0.97–1.02, *p* = 0.41; tegoprazan: RR: 0.99, 95% CI: 0.94–1.05; keverprazan: RR: 1.05, 95% CI: 0.96–1.15, *p* = 0.09) (Supplementary Figure S6.4).

In subgroup analyses base on the types of P-CAB for TEAEs, There was no significant difference in the risk of TEAEs among vonoprazan 10 mg (RR: 0.99, 95% CI: 0.92–1.07, *p* = 0.17) or 20 mg (RR: 1.02, 95% CI: 0.93–1.11, *p* = 0.04), tegoprazan 50 mg (RR: 0.71, 95% CI: 0.41–1.21) or 100 mg (RR: 0.90, 95% CI: 0.55–1.48), keverprazan 20 mg (RR: 0.68, 95% CI: 0.48–97) or 30 mg (RR: 0.99, 95% CI: 0.95–1.14, *p* = 0.91), and lansoprazole (Supplementary Figure S5.5).

### Sensitivity analysis

The overall heterogeneity (I^2^ = 38.32%) of the pooled result was considered moderate. To assess the impact of individual trials on the outcomes, we conducted a sensitivity analysis using the leave-one-out method. This analysis involved the exclusion of one study at a time, followed by the consolidation of data from the remaining studies. As illustrated in Figure 6, regardless of which study was excluded, the lower limit of the 95% CI for the pooled RD of the remaining studies consistently remained within the predefined non-inferiority margin of −6%, affirming the stability of the non-inferiority results. (Supplementary Material S7) displayed the heterogeneity after merging the remaining studies when one study was omitted. It is noteworthy that the removal of Nian-di Tan (2022) led to a substantial reduction in heterogeneity, with the I^2^ decreasing from 38.32% to 0%.

The Galbraith plot and the L’Abbé plot also indicated a high level of heterogeneity associated with the study conducted by Nian-di Tan (2022), because the corresponding point of Nian-di Tan (2022) significantly deviates from the regression line (Supplementary Material S7).

## Disscussion

Over the past 30 years, the introduction of PPI has revolutionized the treatment of acid-related disorders, including PU. However, PPI come with inherent limitations that lead to variability in their acid-suppressing effects (Scarpignato et al., 2016). To address these drawbacks, novel drugs known as P-CABs have been developed. The guidelines in Japan and China recommend PPI and P-CAB as first-line treatments for PU. However, for the prevention of NSAID-induced ulcer, only PPI is recommended (Melcarne, García-Iglesias, and Calvet, 2016; Kamada et al., 2021; Digestion, 2023). It is widely accepted that the enhancement of PU treatment is achieved through the suppression of gastric acid secretion, with superior efficacy attained when sustaining an intragastric pH > 3 for as long as possible within a 24-h period (Howden, Burget, and Hunt, 1994; Schubert and Peura, 2008). Previous pharmacodynamic studies have affirmed that vonoprazan (Echizen, 2016; Scarpignato et al., 2023), tegoprazan (Han et al., 2019), and keverprazan (Li R 2020) are more effective than lansoprazole in maintaining intragastric pH > 3 for this extended duration.

In this meta-analysis, the non-inferiority of P-CABs, which includes vonoprazan, tegoprazan, and keverprazan, when compared to lansoprazole in terms of endoscopic healing for both DU and GU following the standard treatment duration (6 weeks for DU and 8 weeks for GU) was verified, and the tolerability of P-CABs was similar to that of lansoprazole. Additionally, non-inferiority was observed in the effectiveness of P-CABs in preventing NSAID-related ulcer when compared to lansoprazole. Concomitant P-CAB therapy was well tolerated in patients requiring long-term (24 weeks) treatment with NSAIDs, including low-dose aspirin for the prevention of cerebrovascular events, and in those necessitating NSAID therapy for pain management. Although our study observed a slightly higher incidence of serious adverse events associated with P-CABs compared to lansoprazole, it is essential to emphasize that these serious adverse events related to the study drugs were infrequent (P-CAB: 9.6% and lansoprazole: 9.3%). Serious adverse events unrelated to the study drugs including acute pancreatitis, subcutaneous abscess, and muscular weakness, among others. This result was consistent with the previously meta-analysis about adverse events of vonoprazan (Xu et al., 2023). Further investigation is needed to determine whether these events are indeed related to the use of P-CABs.

Our subgroup analyses confirmed the non-inferiority of P-CABs compared to lansoprazole, except for the 4-week healing rate of GU. It is crucial to provide a meticulous explanation for this result. As presented in Table 1, the non-inferiority margin was 6% for DU, and the minimum non-inferiority margin for GU was 8%. However, when we redefined with an 8% margin for GU, the lower limit of the 95% CI for RD (−6.6%) falls within the −8% margin. This suggests that P-CABs could still be considered non-inferior in the treatment of GU at 4 weeks, and the observed difference may be attributed to our non-inferiority margin being overly conservative.

In sensitivity analyses, we identified that the primary source of heterogeneity originated from the Nian-di Tan (2022)’ s study. After a thorough comparison with the other studies, we have summarized that the sources of heterogeneity came from three aspects: First, the sample size in Nian-di Tan (2022)’ s study was notably smaller than the other four studies, potentially resulting in a small sample study effect. Second, the patient withdrawal rate in Nian-di Tan (2022)’ s study is the highest among the five studies, which may lead to attrition bias. Third, there was methodological heterogeneity in Nian-di Tan (2022)’ s study, as RD and non-inferiority margin have not been reported.

Based on our findings, we have offered several recommendations for future research on P-CABs. First, while current research has mainly focused on Asian populations, necessitating further evaluation of its efficacy and safety in diverse demographic groups. Second, the long-term use of PPIs for gastric acid inhibition has been associated with various adverse events, such as fractures (Yu et al., 2011), chronic kidney disease (Wu et al., 2023), and *Clostridium difficile* infection (Deshpande et al., 2012). Given that P-CABs exhibit more potent acid inhibition compared to PPIs, investigating whether they are more likely to result in long-term adverse events requires further observational studies from real-world. Third, as PPIs compete to inhibit the CYP2C19 enzyme, thereby reducing the antiplatelet effect of clopidogrel (Norgard, Mathews, and Wall, 2009), it is crucial to explore whether vonoprazan, which also interacts with CYP2C19, affects antiplatelet efficacy in patients requiring long-term antiplatelet therapy, potentially leading to an increased risk of cardiovascular adverse events. Fourth, future research should address whether P-CABs can be effectively utilized for the prevention of stress ulcers or the treatment of idiopathic ulcers. Fifth, future research should also compare P-CABs with PPIs other than lansoprazole.

Our meta-analysis is currently the first comprehensive study to evaluate the efficacy and safety of P-CABs in the treatment of PU, we did a comprehensive systematic review and included seven RCTs with high-quality. All included studies were randomized, double-blind, parallel controlled, and multi-center trials, all of which clearly reported the methods of randomization and allocation concealment, while strictly following the reporting requirements of CONSORT (Piaggio et al., 2012). Our analysis not only included an assessment of superiority using RR but also evaluated non-inferiority through RD. In fact, given the substantial performance of lansoprazole in treating peptic ulcers, conducting a non-inferiority analysis is more rational and rigorous (Acuna, Dossa, and Baxter, 2020). Furthermore, we conducted a comprehensive set of subgroup analyses to thoroughly assess the effectiveness and safety of P-CABs in PU treatment.

This study has some limitations. First, we did not conduct a subgroup analysis based on *H. pylori* status, as Hou (2022) did not report the relevant data. We attempted to require relevant data from the author via email but did not receive a response. We plan to update the results once we obtain the necessary data. Second, the evaluation of P-CABs for symptom relief was challenging due to inconsistencies in the original studies regarding the endpoints of symptom remission. Some studies used frequency, some used scores, and others used remission rates. Third, due to the limited number of included studies, we were unable to conduct a more comprehensive dose-response analysis of P-CABs. Fourth, we restricted our meta-analysis to studies published in English due to the notable risk of bias identified in the Chinese studies we examined, which may lead to potential language bias.

In conclusion, P-CABs was non-inferior to lansoprazole in healing PU after 4–8 weeks treatment and prevention of PU recurrence during NSAID therapy. The safety profile of P-CABs was similar to that of lansoprazole. However, P-CABs had higher incidences of the serious adverse events compared to lansoprazole.

## Data Availability

The original contributions presented in the study are included in the article/Supplementary Material, further inquiries can be directed to the corresponding author.

## Abbreviations

AE, adverse event; CI, confidence interval; CONSORT, Consolidated Standards of Reporting Trials; DU, duodenal ulcer; ESD, endoscopic submucosal dissection; FAS, full analysis set; GU, gastric ulcer; *H. pylori*, *H.elicobacter pylori*; NSAID, Non-steroidal anti-inflammatory drug; P-CAB, potassium competitive acid blocker; PP, per protocol; PPI, proton pump inhibitor; PRISMA, Preferred Reporting Items for Systematic Reviews and Meta-Analyses; PU, peptic ulcer; RCTs, randomized controlled trials; RD, rate difference; RR, risk ratio; TEAEs, treatment-emergent adverse events.
