# Supplementary material for: Comparative efficiency and safety of potassium competitive acid blockers versus Lansoprazole in peptic ulcer: a systematic review and meta-analysis
Source: Front Pharmacol. 2024 Jan 11;14:1304552. doi: 10.3389/fphar.2023.1304552 (PMC10808560; doi:10.3389/fphar.2023.1304552)
Supplement: Supplementary file 1 [file DataSheet1.docx]

**Contents** **of** **Supplementary** **appendix**

| Supplementary 1 | Published protocol and changes made to the protocol | Page 2 |
| --- | --- | --- |
| Supplementary 2 | Search strategy and results | Page 4 |
| Supplementary 3 | References for included trials | Page 5 |
| Supplementary 4 | Extracted original data for each outcome | Page 6 |
| Supplementary 5 | Subgroup meta-analyses for primary outcomes | Page 8 |
| Supplementary 6 | Pooled risk ratios for primary outcomes | Page 13 |
| Supplementary 7 | Sensitivity meta-analyses | Page 16 |
| Supplementary 8 | PRISMA checklist | Page 19 |

**Supplementary 1**

**Published protocol and changes made to the protocol**

The protocol has been registered in PROSPERO (No. CRD42023458361) and it was available at [https://www.crd.york.ac.uk/PROSPERO/display_record.php?RecordID=458361](mailto:https:/www.crd.york.ac.uk/PROSPERO/display_record.php?RecordID=458361)

**Here** **below** **some** **changes** **and** **clarifications** **to** **the** **published** **protocol:**

1.In all the included studies, the control group was lansoprazole. Unfortunately, we were unable to locate any comparative data between Potassium Competitive Acid Blockers (P-CABs) and other Proton Pump Inhibitors (PPIs) in the context of peptic ulcers. Therefore, we have made the decision to revise the title of our review from "Comparative Efficiency and Safety of Potassium Competitive Acid Blockers Versus Proton Pump Inhibitors in Peptic Ulcer: A Systematic Review and Meta-Analysis" to "Comparative Efficiency and Safety of Potassium Competitive Acid Blockers Versus Lansoprazole in Peptic Ulcer: A Systematic Review and Meta-Analysis."

2.Following a comprehensive search of Chinese databases (such as VIP, CNKI, Wanfang, etc.), we identified three Chinese studies that met our inclusion criteria. However, these studies exhibited substantial limitations in sample size, research design, methodology, and outcome assessment, potentially causing significant bias into our meta-analyses. Therefore, we did not include Chinese study.

3.All the studies included in our review were non-inferiority trials, and none of them could prove the superiority of PCABs over lansoprazole in the treatment of ulcers. Given that lansoprazole already demonstrates substantial efficacy in ulcer management, the focus of our analysis shifted to non-inferiority assessments. From a statistical perspective, we calculated the risk ratio (RR) according to the protocol settings, this metric is primarily applied in studies designed for superiority analysis and may not suitable for non-inferiority trials. Consequently, we extracted non-inferiority margins from all the included studies and opted for the most conservative margin to ensure the interpretability of non-inferiority. The validity of non-inferiority when the lower 95% confidence interval (95% CI) of the pooled risk difference fell within these non-inferiority margins.


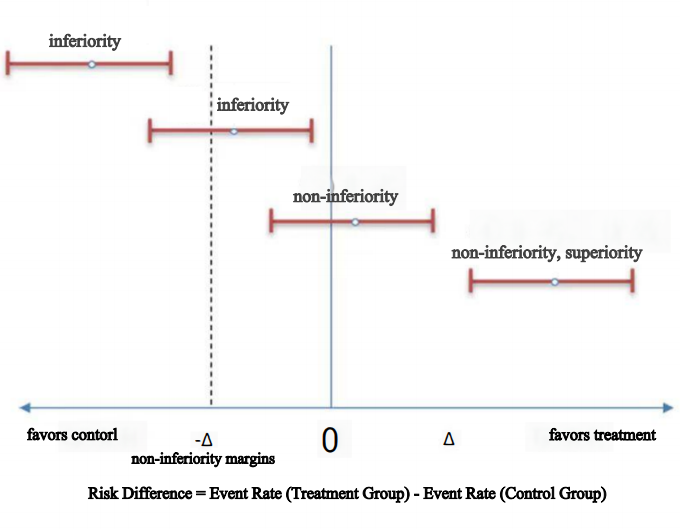


4.In the sensitivity analysis, we added Galbraith and L'Abbé plots to find the sources of heterogeneity within our results.

**Supplementary 2**

**Search strategy and results**

**Number** **of** **citations** **by** **each** **database** **searched**

| **Databases:** | **Citations** |
| --- | --- |
| PubMed | 181 |
| Cochrane | 110 |
| Embase | 402 |
| Web of Science | 146 |
| Scopus | 331 |
| **Total** **(databases)** | **1170** |

**PUBMED**

((((((((((proton pump inhibitor) OR (ppi)) OR (omeprazole)) OR (esomeprazole)) OR (dexlansoprazole)) OR (lansoprazole)) OR (pantoprazole)) OR (rabeprazole)) OR (ilaprazole)) AND (((((((potassium competitive acid blocker) OR (PCAB)) OR (vonoprazan)) OR (tegoprazan)) OR (fexuprazan)) OR (revaprazan)) OR (keverprazan))) AND ((((((((ulcer) OR (peptic ulcer)) OR (gastric ulcer)) OR (duodenal ulcer)) OR (drug-induced ulcer)) OR (idiopathic ulcer)) OR (NSAID-induced ulcer)) OR (LDA-induced ulcer))

**181**

**WEB OF SCIENCE**

#1：(TS=(potassium competitive acid blocker OR PCAB OR vonoprazan OR tegoprazan OR fexuprazan OR revaprazan OR keverprazan OR soraprazan OR linaprazan)) AND language: (English): 1222

#2：(TS=(proton pump inhibitor OR ppi OR omeprazole OR esomeprazole OR dexlansoprazole OR lansoprazole OR pantoprazole OR rabeprazole OR ilaprazole) )  AND language: (English): 62013

#3: (TS=(ulcer OR peptic ulcer OR gastric ulcer OR duodenal ulcer OR drug-induced ulcer OR idiopathic ulcer OR NSAID-induced ulcer OR LDA-induced ulcer

) )  AND language: (English): 124501

#4 #1AND #2AND#3 **146**

**COCHRANE**

#1 potassium competitive acid blocker OR PCAB OR vonoprazan OR tegoprazan OR fexuprazan OR revaprazan OR keverprazan OR soraprazan OR linaprazan:ab,ti,kw : #2 proton pump inhibitor OR ppi OR omeprazole OR esomeprazole OR dexlansoprazole OR lansoprazole OR pantoprazole OR rabeprazole OR ilaprazole:ab,ti,kw:

#3 ulcer OR peptic ulcer OR gastric ulcer OR duodenal ulcer OR drug-induced ulcer OR idiopathic ulcer OR NSAID-induced ulcer OR LDA-induced ulcer :ab,ti,kw:

#4：#1 AND #2 AND #3 : **110**Trials

**EMBASE**

#1 ('potassium'/exp OR potassium) AND competitive AND ('acid'/exp OR acid) AND blocker OR pcab OR 'vonoprazan'/exp OR vonoprazan OR 'tegoprazan'/exp OR tegoprazan OR 'fexuprazan'/exp OR fexuprazan OR 'revaprazan'/exp OR revaprazan OR keverprazan OR 'soraprazan'/exp OR soraprazan OR 'linaprazan'/exp OR linaprazan [1,811](https://www.embase.com/)

#2'proton pump inhibitor'/exp OR 'proton pump inhibitor' OR ppi OR 'omeprazole'/exp OR omeprazole OR 'esomeprazole'/exp OR esomeprazole OR 'dexlansoprazole'/exp OR dexlansoprazole OR 'lansoprazole'/exp OR lansoprazole OR 'pantoprazole'/exp OR pantoprazole OR 'rabeprazole'/exp OR rabeprazole OR 'ilaprazole'/exp OR ilaprazole

[129,645](https://www.embase.com/)

#3((((((('ulcer'/exp OR ulcer OR peptic) AND ('ulcer'/exp OR ulcer) OR gastric) AND ('ulcer'/exp OR ulcer) OR duodenal) AND ('ulcer'/exp OR ulcer) OR 'drug induced') AND ('ulcer'/exp OR ulcer) OR idiopathic) AND ('ulcer'/exp OR ulcer) OR 'nsaid induced') AND ('ulcer'/exp OR ulcer) OR 'lda induced') AND ('ulcer'/exp OR ulcer) 357,410

#4 #1 AND #2 AND #3  **402**

**[SCOPUS](http://www.vziliao.com/Entrance/Open/ba6c3dba-c012-440f-b7e9-33d2a53c7ae6)**

( TITLE-ABS-KEY ( potassium  AND competitive  AND acid  AND blocker  OR  pcab  OR  vonoprazan  OR  tegoprazan  OR  fexuprazan  OR  revaprazan  OR  keverprazan  OR  soraprazan  OR  linaprazan )  AND  TITLE-ABS-KEY ( proton  AND pump  AND inhibitor  OR  ppi  OR  omeprazole  OR  esomeprazole  OR  dexlansoprazole  OR  lansoprazole  OR  pantoprazole  OR  rabeprazole  OR  ilaprazole )  AND  TITLE-ABS-KEY ( ulcer  OR  peptic  AND ulcer  OR  gastric  AND ulcer  OR  duodenal  AND ulcer  OR  drug-induced  AND ulcer  OR  idiopathic  AND ulcer  OR  nsaid-induced  AND ulcer  OR  lda-induced  AND ulcer ) )  **331**

**Supplementary 3**

**References for included trials**

1. Miwa H, Uedo N, Watari J, Mori Y, Sakurai Y, Takanami Y, et al. Randomised clinical trial: efficacy and safety of vonoprazan vs. lansoprazole in patients with gastric or duodenal ulcers – results from two phase 3, non-inferiority randomised controlled trials. Alimentary Pharmacology and Therapeutics. 2017;45(2):240-52.

2. Kawai T, Oda K, Funao N, Nishimura A, Matsumoto Y, Mizokami Y, et al. Vonoprazan prevents low-dose aspirin-associated ulcer recurrence: randomised phase 3 study. Gut. 2018;67(6):1033-41.

3. Mizokami Y, Oda K, Funao N, Nishimura A, Soen S, Kawai T, et al. Vonoprazan prevents ulcer recurrence during long-term NSAID therapy: randomised, lansoprazole-controlled non-inferiority and single-blind extension study. Gut. 2018;67(6):1042-51.

4. Cho YK, Choi MG, Choi SC, Lee KM, Kim TO, Park SH, et al. Randomised clinical trial: tegoprazan, a novel potassium-competitive acid blocker, or lansoprazole in the treatment of gastric ulcer. Alimentary pharmacology & therapeutics. 2020;52(5):789-97.

5. Hou X, Meng F, Wang J, Sha W, Chiu CT, Chung WC, et al. Vonoprazan non-inferior to lansoprazole in treating duodenal ulcer and eradicating Helicobacter pylori in Asian patients. Journal of gastroenterology and hepatology. 2022;37(7):1275-83.

6. Tan N, Liu XW, Liu CX, Li SB, Chen HH, Li X, et al. The efficacy of keverprazan for duodenal ulcer: a phase II randomized, double-blind, parallel-controlled trial. Journal of gastroenterology and hepatology. 2022.

7. Tan N, Miao XP, Liao AJ, Liu CX, Wu H, Chen HH, et al. Efficacy and safety of keverprazan compared to lansoprazole in the treatment of duodenal ulcer: a phase III, randomised, double-blind, multicentre trial. Clinical and translational gastroenterology. 2023.

**Supplementary 4**

**Extracted original data for each outcome**

| **Primary outcomes** | | | | | |
| --- | --- | --- | --- | --- | --- |
| **Healing rate of peptic ulcer** | | | | | |
| Study | Yes | No | Yes | No |  |
| H.Miwa(2016) | 386 | 23 | 388 | 17 |  |
| Yu Kyung Cho(2020) | 185 | 10 | 89 | 4 |  |
| Nian-di Tan(2022) | 113 | 3 | 56 | 8 |  |
| Hou(2022) | 246 | 8 | 246 | 9 |  |
| Nian-di Tan(2023) | 170 | 10 | 166 | 12 |  |
|  |  |  |  |  |  |
| **Subgroup analysis based on ulcer location** | | | | | |
| Study | Yes | No | Yes | No | subgroup |
| H.Miwa(2016) | 170 | 8 | 177 | 3 | DU |
| Nian-di Tan(2022) | 113 | 3 | 56 | 8 | DU |
| Hou(2022) | 246 | 8 | 246 | 9 | DU |
| Nian-di Tan(2023) | 170 | 10 | 166 | 12 | DU |
| H.Miwa(2016) | 216 | 15 | 211 | 14 | GU |
| Yu Kyung Cho(2020) | 185 | 10 | 89 | 4 | GU |
|  |  |  |  |  |  |
| **Subgroup analysis based on the follow-up time for GU** | | | | | |
| Study | Yes | No | Yes | No | subgroup |
| H.Miwa(2016) | 169 | 62 | 170 | 55 | 4week |
| Yu Kyung Cho(2020) | 178 | 17 | 83 | 10 | 4week |
| H.Miwa(2016) | 216 | 15 | 211 | 14 | 8week |
| Yu Kyung Cho(2020) | 185 | 10 | 89 | 4 | 8week |
|  |  |  |  |  |  |
| **Subgroup analysis based on the follow-up time for DU** | | | | | |
| Study | Yes | No | Yes | No | subgroup |
| H.Miwa(2016) | 167 | 11 | 170 | 10 | 4week |
| Nian-di Tan(2022) | 103 | 13 | 51 | 13 | 4week |
| Hou(2022) | 222 | 27 | 222 | 29 | 4week |
| Nian-di Tan(2023) | 151 | 29 | 143 | 35 | 4week |
| H.Miwa(2016) | 170 | 8 | 177 | 3 | 6Week |
| Nian-di Tan(2022) | 113 | 3 | 56 | 8 | 6Week |
| Hou(2022) | 246 | 8 | 246 | 9 | 6Week |
| Nian-di Tan(2023) | 170 | 10 | 166 | 12 | 6Week |
|  |  |  |  |  |  |
| **Subgroup analysis based on the types of P-CAB** | | | | | |
| Study | Yes | No | Yes | No | subgroup |
| H.Miwa(2016) | 386 | 23 | 388 | 17 | Vonoprazan |
| Yu Kyung Cho(2020) | 185 | 10 | 89 | 4 | Tegoprazan |
| Nian-di Tan(2022) | 113 | 3 | 56 | 8 | keverprazan |
| Hou(2022) | 246 | 8 | 246 | 9 | Vonoprazan |
| Nian-di Tan(2023) | 170 | 10 | 166 | 12 | keverprazan |
| **Secondary outcomes** | | | | | |
| **Recurrence rates of drug-related peptic ulcers** | | | | | |
| Study | Yes | No | Yes | No | subgroup |
| Kawai(2018) | 2 | 391 | 2 | 211 | 12Week |
| Mizokami(2018) | 12 | 400 | 10 | 189 | 12Week |
| Kawai(2018) | 4 | 389 | 6 | 207 | 24week |
| Mizokami(2018) | 14 | 398 | 11 | 188 | 24week |
|  |  |  |  |  |  |
| **Safety assessments of treatment-emergent** **adverse events(TEAEs)** | | | | | |
| Study | Yes | No | Yes | No |  |
| H.Miwa(2016) | 128 | 299 | 132 | 291 |  |
| Kawai(2018) | 359 | 71 | 184 | 33 |  |
| Mizokami(2018) | 353 | 51 | 185 | 25 |  |
| Yu Kyung Cho(2020) | 41 | 163 | 25 | 75 |  |
| Nian-di Tan(2022) | 63 | 53 | 41 | 23 |  |
| Hou(2022) | 195 | 68 | 174 | 94 |  |
| Nian-di Tan(2023) | 104 | 76 | 105 | 73 |  |
|  |  |  |  |  |  |
| **Safety assessments of serious adverse events** | | | | | |
| Study | Yes | No | Yes | No |  |
| H.Miwa(2016) | 12 | 415 | 8 | 415 |  |
| Kawai(2018) | 65 | 339 | 32 | 185 |  |
| Mizokami(2018) | 48 | 382 | 18 | 192 |  |
| Yu Kyung Cho(2020) | 5 | 199 | 1 | 99 |  |
| Nian-di Tan(2022) | 1 | 115 | 2 | 64 |  |
| Hou(2022) | 11 | 252 | 2 | 266 |  |
| Nian-di Tan(2023) | 3 | 177 | 1 | 177 |  |
|  |  |  |  |  |  |
| **Subgroup analysis based on the types of P-CAB for TEAEs** | | | | | |
| Study | Yes | No | Yes | No | subgroup |
| H.Miwa(2016) | 128 | 299 | 132 | 291 | Vonoprazan20mg |
| Kawai(2018) | 177 | 25 | 184 | 33 | Vonoprazan10mg |
| Kawai(2018) | 176 | 26 | 184 | 33 | Vonoprazan20mg |
| Mizokami(2018) | 184 | 34 | 185 | 25 | Vonoprazan10mg |
| Mizokami(2018) | 175 | 37 | 185 | 25 | Vonoprazan20mg |
| Yu Kyung Cho(2020) | 18 | 84 | 25 | 75 | Tegoprazan50mg |
| Yu Kyung Cho(2020) | 23 | 79 | 25 | 75 | Tegoprazan100mg |
| Nian-di Tan(2022) | 24 | 31 | 41 | 23 | keverprazan20mg |
| Nian-di Tan(2022) | 39 | 22 | 41 | 23 | keverprazan30mg |
| Hou(2022) | 195 | 68 | 174 | 94 | Vonoprazan20mg |
| Nian-di Tan(2023) | 104 | 76 | 105 | 73 | keverprazan30mg |

**Supplementary 5**

**Subgroup meta-analyses for primary outcomes**

**Figure S5.1 Subgroup analysis based on ulcer location**

**
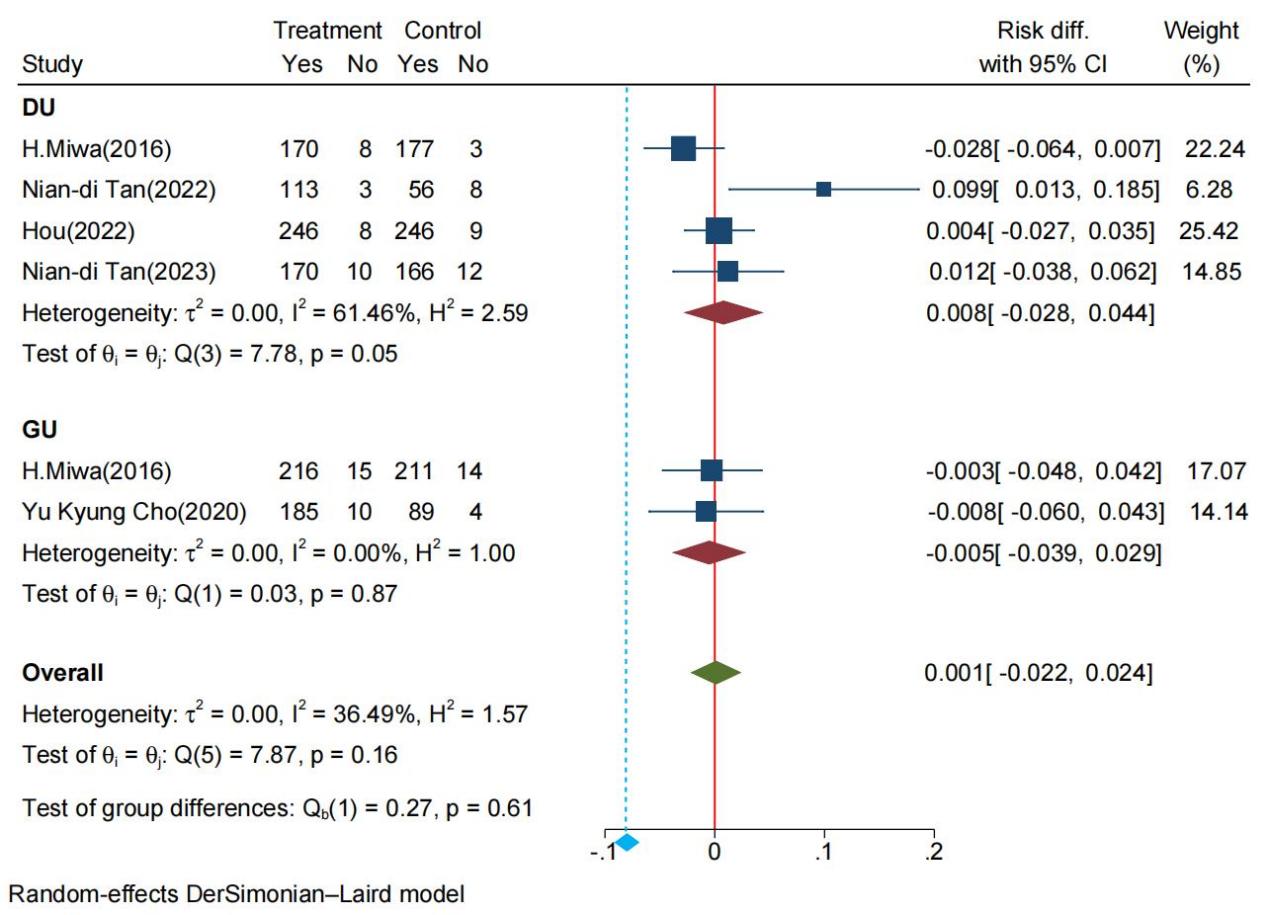
**

**Forrest plot for subgroup analysis based on ulcer location (random-effects model) CI: confidence interval; DU: duodenal ulcer; GU: gastric ulcer.**

**Figure S5.2 Subgroup analysis based on follow-up time for GU or DU**

**
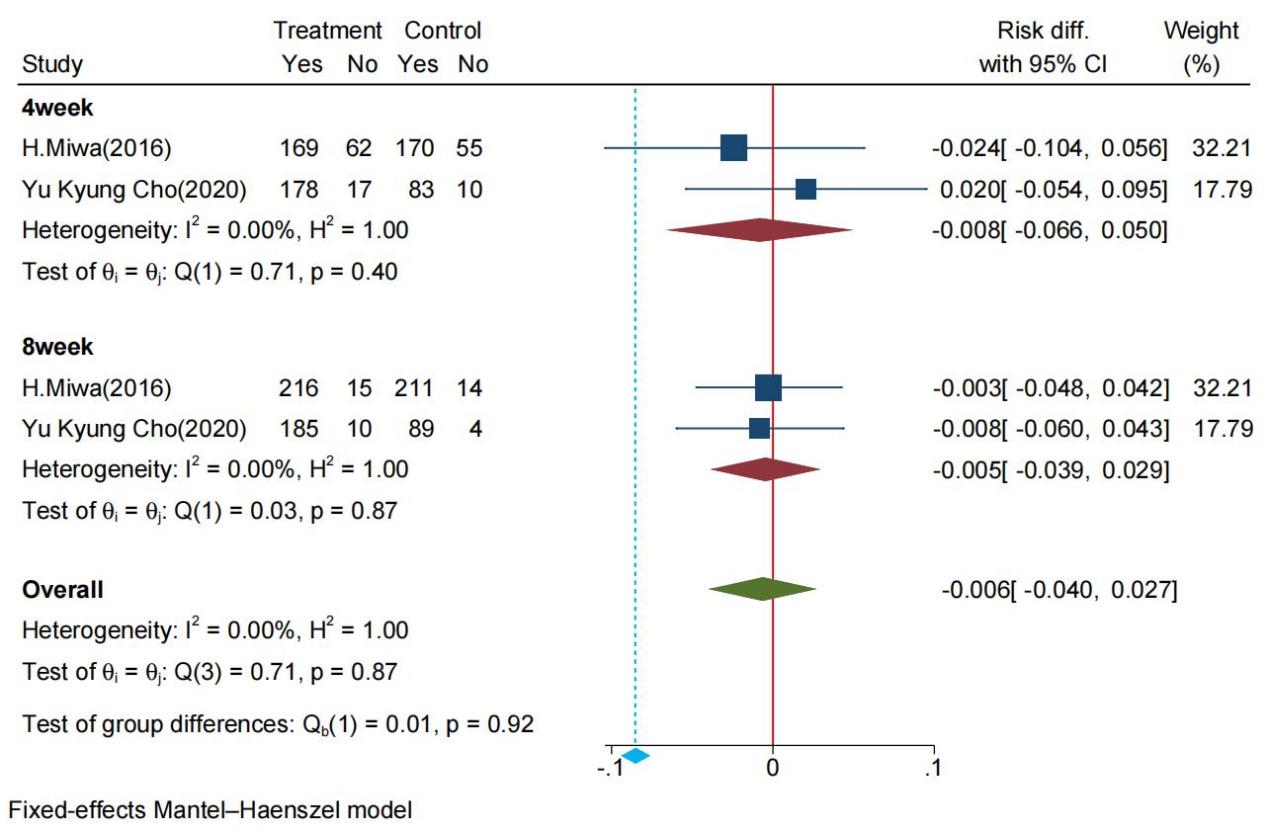
**

**Forrest plot for subgroup analysis based on the follow-up time for GU (fixed-effects model) CI: confidence interval; GU: gastric ulcer.**

**
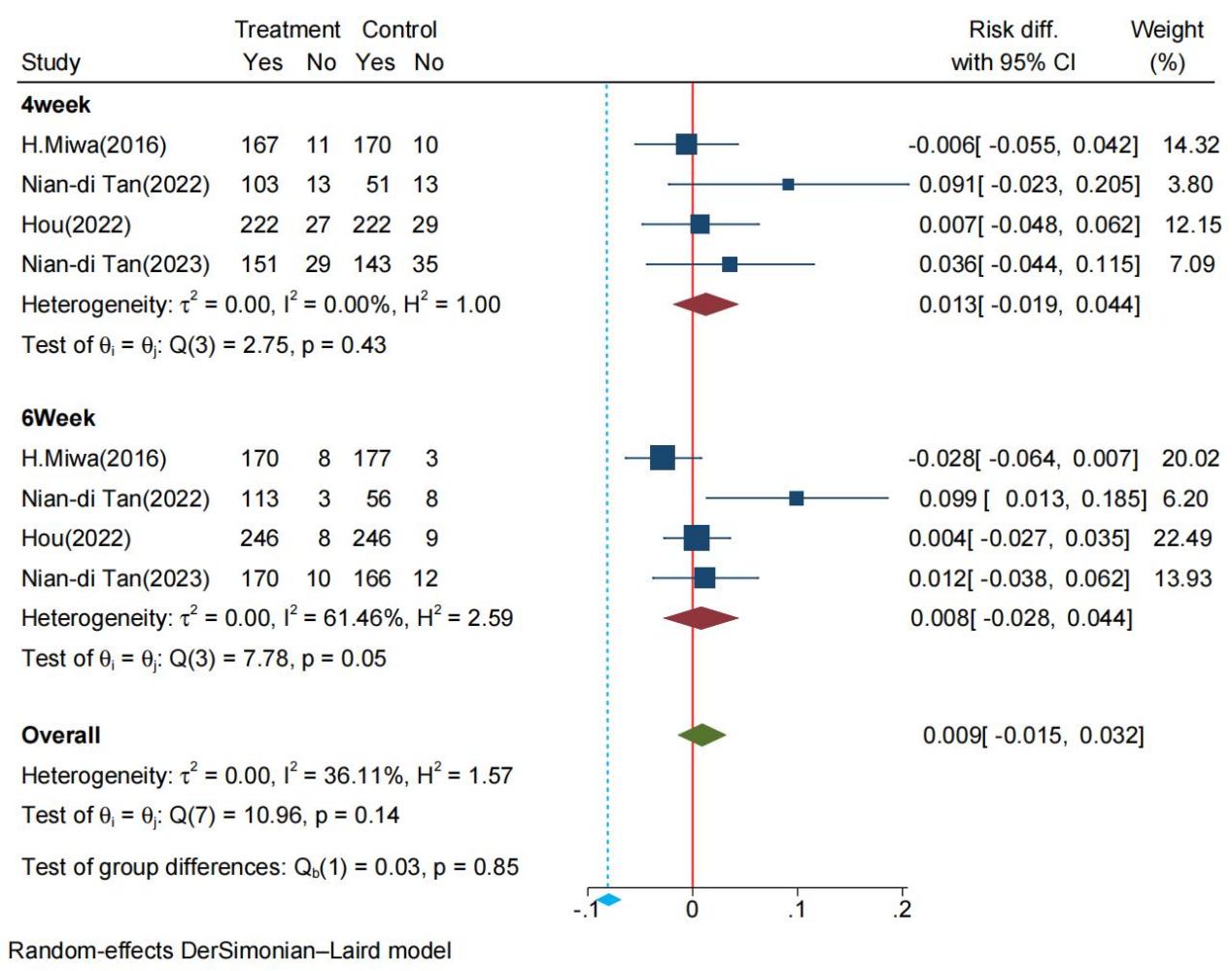
**

**Forrest plot for subgroup analysis based on the follow-up time for DU (random-effects model) CI: confidence interval; DU: duodenal ulcer.**

**Figure S5.3 Subgroup analysis based on the types of P-CAB**

**
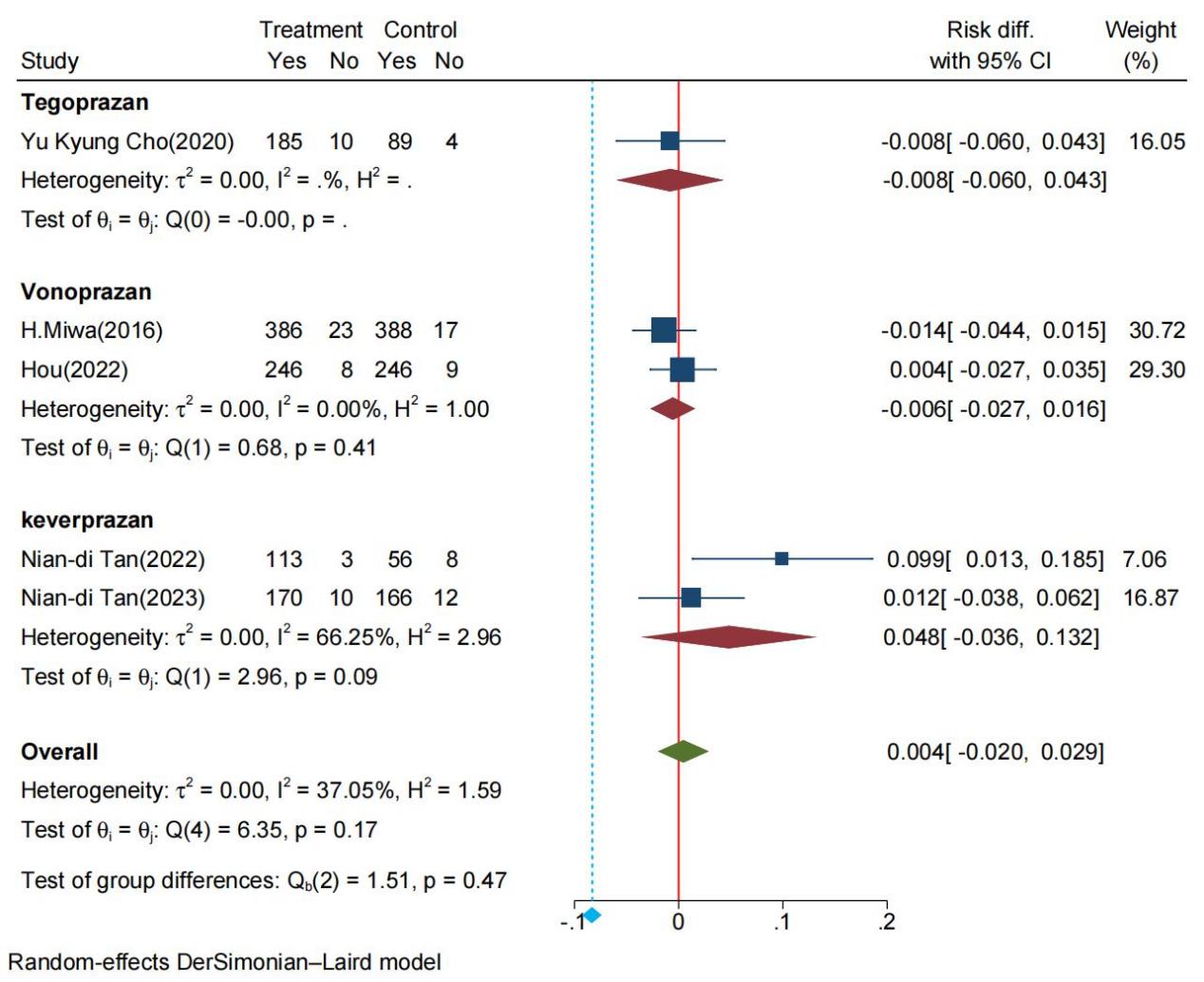
**

**Forrest plot for subgroup analysis based on the types of P-CAB (random-effects model) CI: confidence interval.**

**Figure S5.4 Safety assessments of serious adverse events**

**
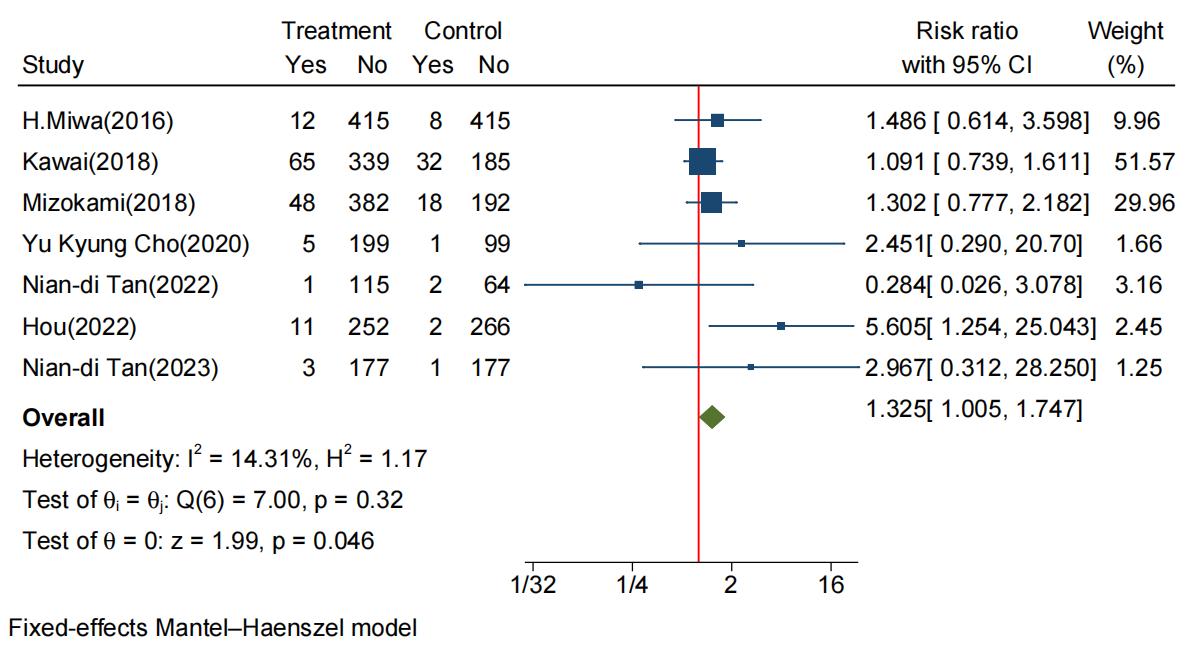
**

**Forrest plot for subgroup analysis for serious adverse events (fixed-effects model) CI: confidence interval.**

**Figure S5.5 Subgroup analysis based on the types of P-CAB for TEAEs**

**
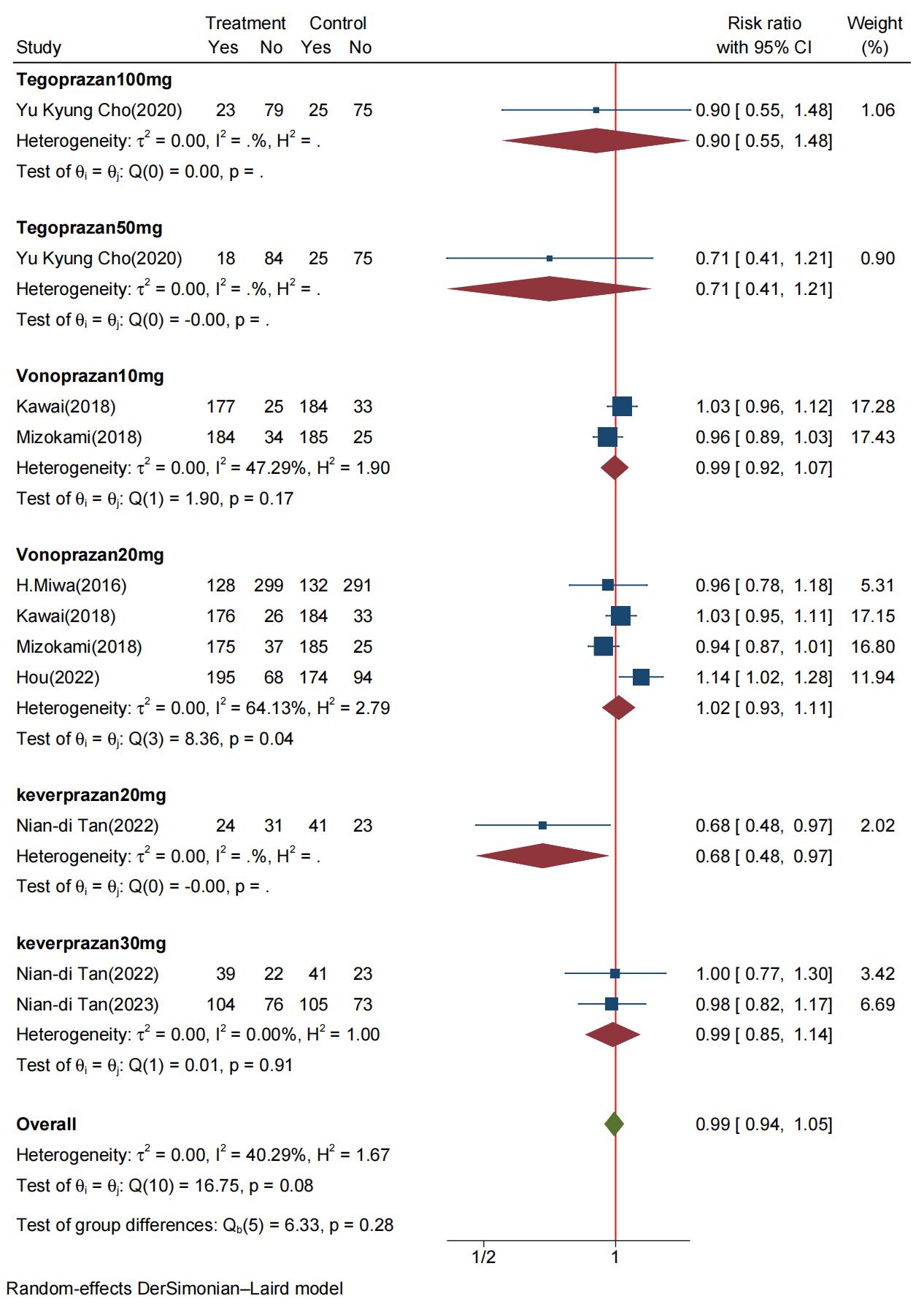
**

**Forrest plot for subgroup analysis for TEAEs based on the types of P-CAB (random-effects model) CI: confidence interval.**

**Supplementary 6**

**Pooled risk ratios for primary outcomes**

**Figure S6.1 The pooled risk ratio(RR) of healing rate in peptic ulcer**

**
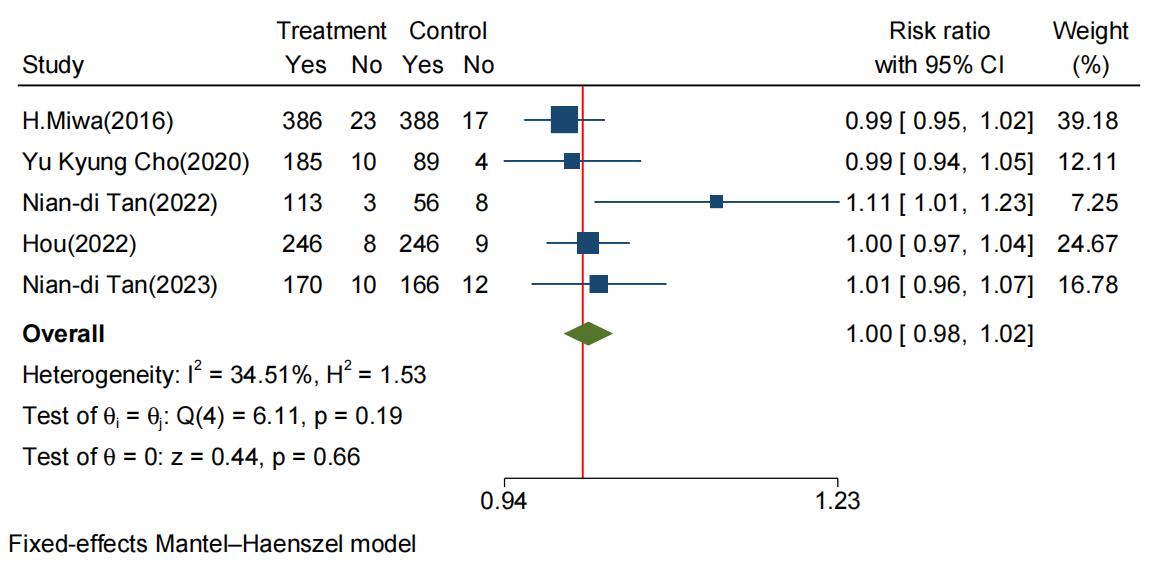
**

**Forrest plot for healing rate of peptic ulcer (fixed-effects model) CI: confidence interval.**

**Figure S6.2 The pooled RR for recurrence rate of NSAID-related ulcer**

**
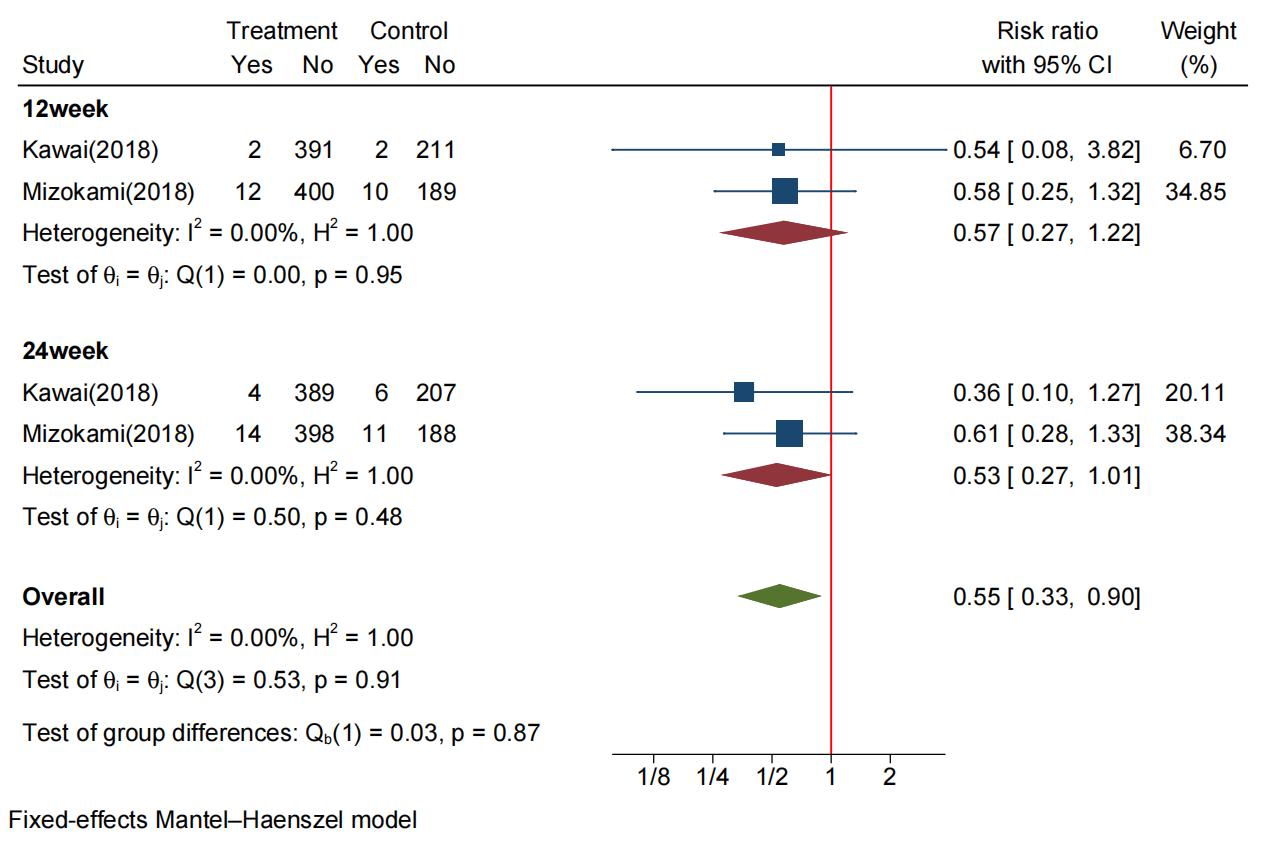
**

**Forrest plot for recurrence rate of NSAID-related ulcer (fixed-effects model)**

**CI: confidence interval.**

**Figure S6.3 Subgroup analysis for RR based on ulcer location**

**
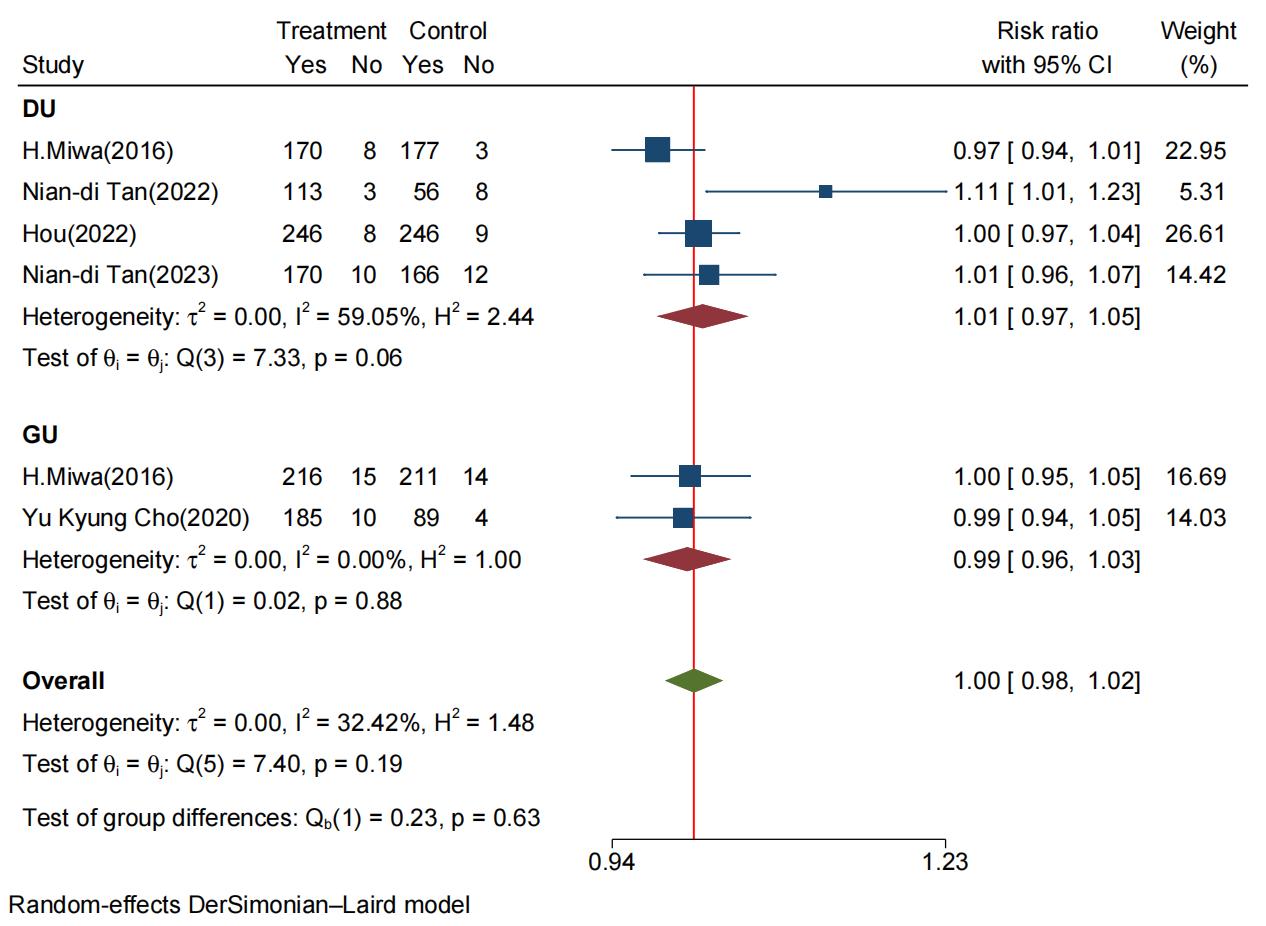
**

**Forrest plot for subgroup analysis based on ulcer location (random-effects model) CI: confidence interval; DU: duodenal ulcer; GU: gastric ulcer.**

**Figure S6.4 Subgroup analysis for RR based on follow-up time of GU or DU**

**
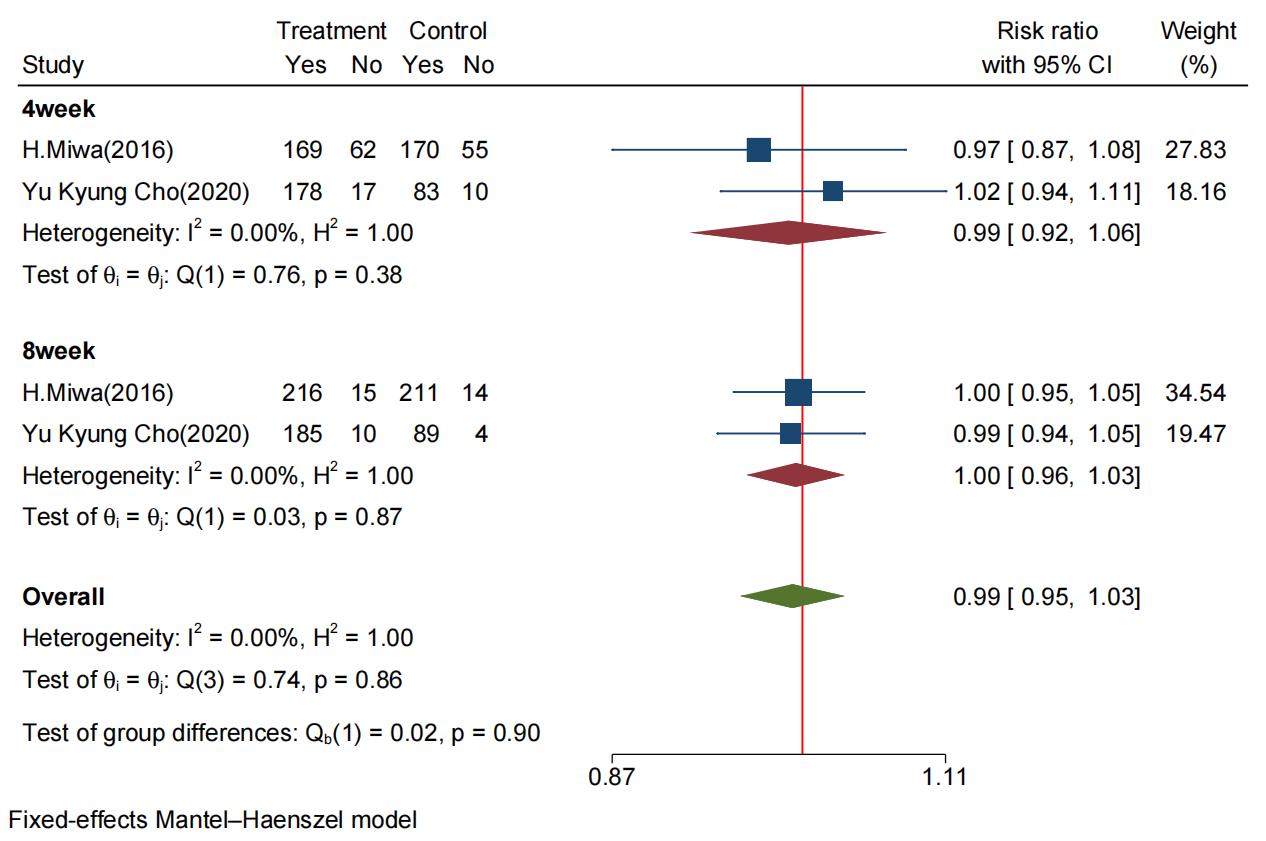
**

**Forrest plot for subgroup analysis based on the follow-up time for GU (fixed-effects model) CI: confidence interval; GU: gastric ulcer.**

**
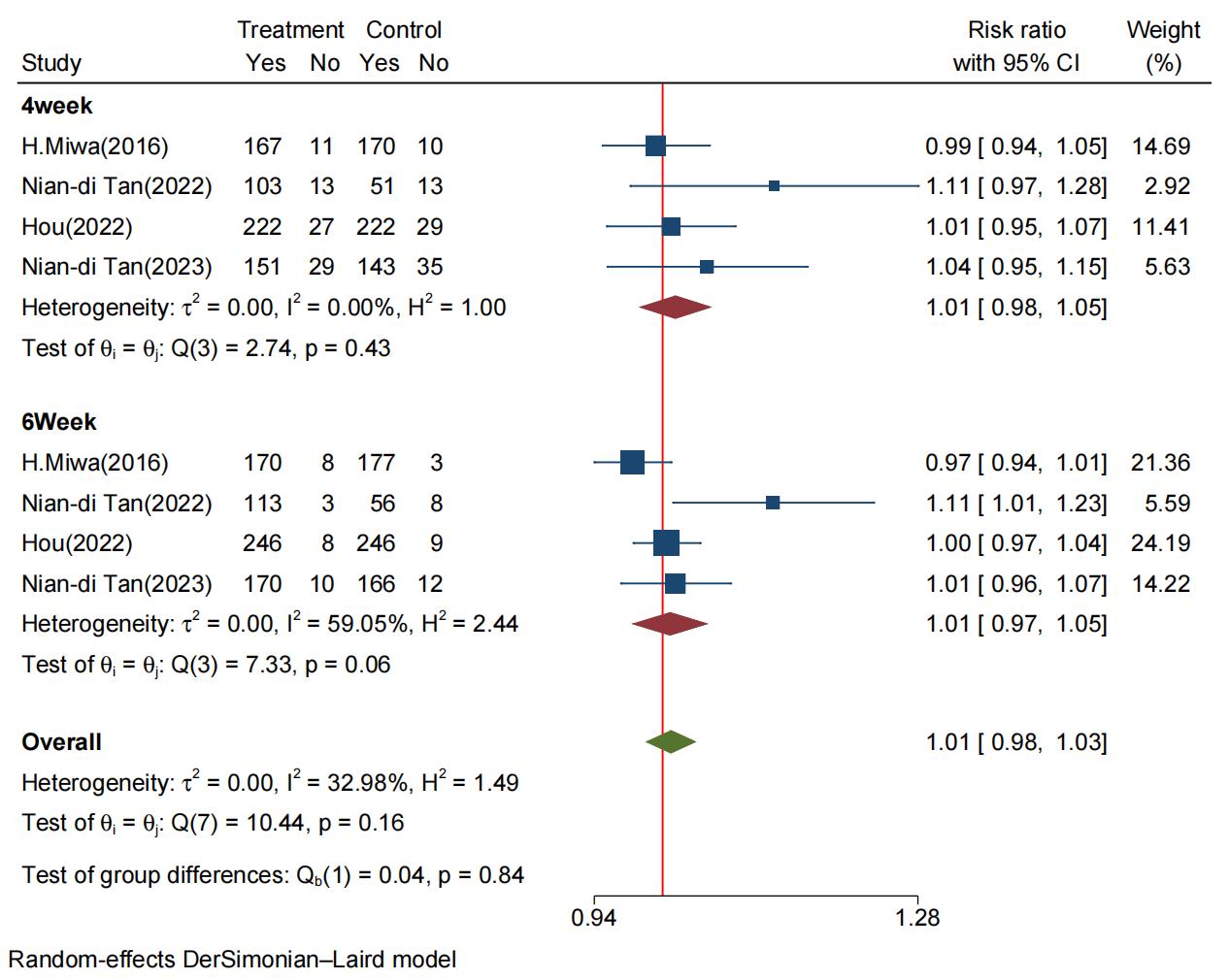
**

**Forrest plot for subgroup analysis based on the follow-up time for DU (random-effects model) CI: confidence interval; DU: duodenal ulcer.**

**Figure S6.5 Subgroup analysis for RR based on the types of P-CAB**

**
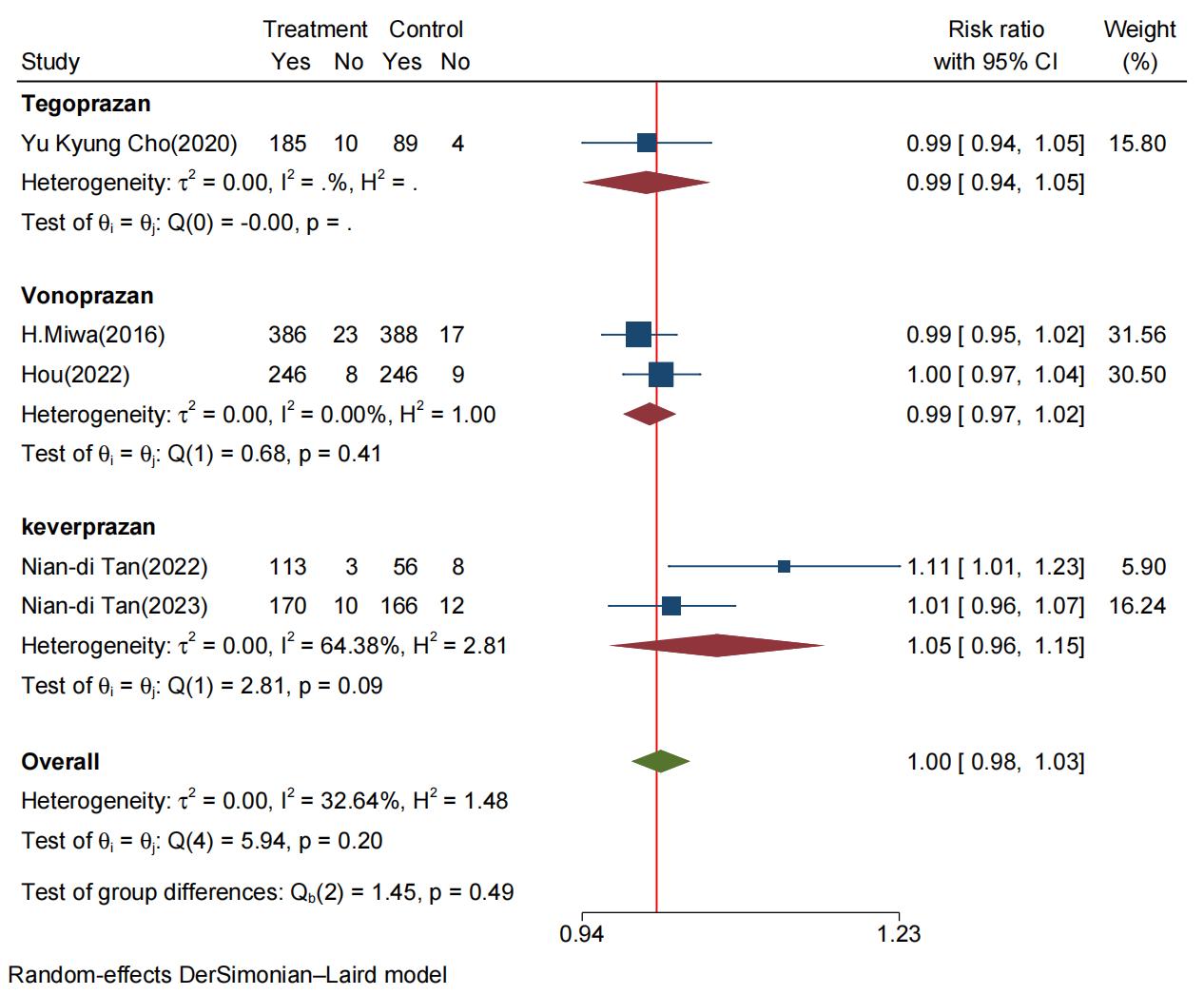
**

**Forrest plot for subgroup analysis based on the types of P-CAB (random-effects model) CI: confidence interval.**

**Supplementary 7**

**Sensitivity meta-analyses**

**Figure S7.1 Sensitivity analysis using the leave-one-out method**


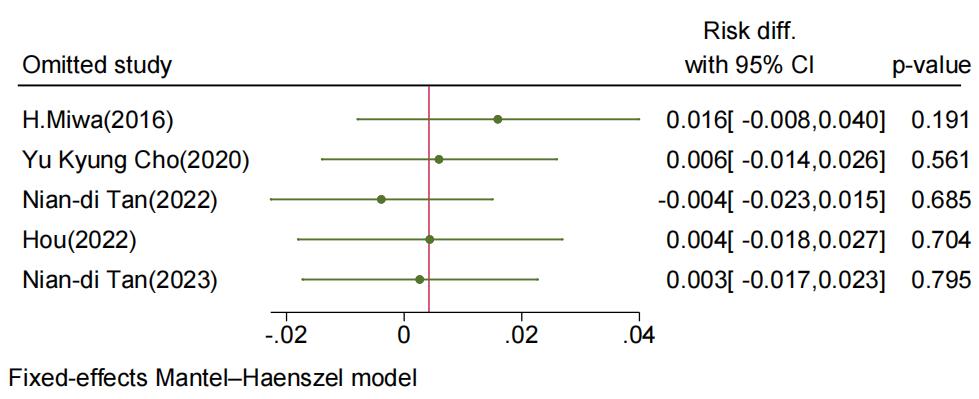


The I^2^ after omitting one study

| **Omitted study** | **I^2^** |
| --- | --- |
| H.Miwa(2016) | 40.56 |
| Yu Kyung Cho(2020) | 52.84 |
| Nian-di Tan(2022) | 0 |
| Hou(2022) | 53.80 |
| Nian-di Tan(2023) | 52.07 |

**Figure S7.2 Sensitivity analysis using the Galbraith plot**


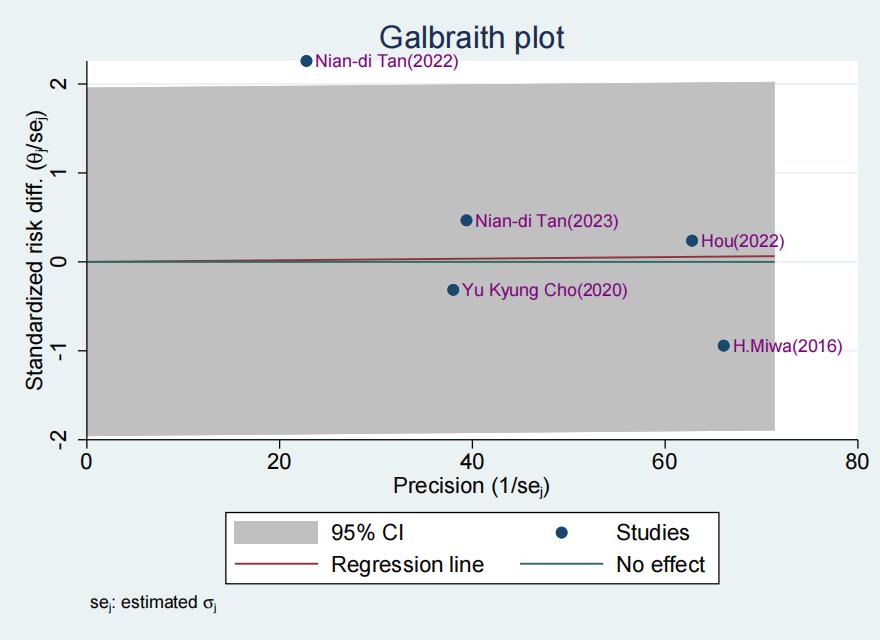


The Galbraith plots are based on the reciprocal of the standardized estimate (such as logOR/ lnRR/ SE) against the standard error. If the scatter slopes are close, it means that the included studies are homogeneous.

**Figure S7.3 Sensitivity analysis using the L’ Abbe plot**


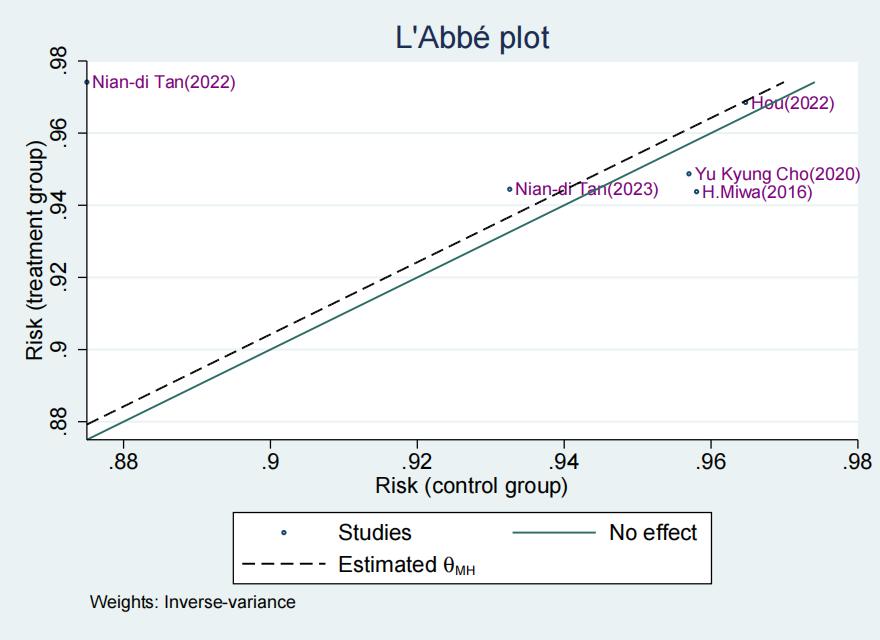


L’ Abbe plots are commonly used to test for heterogeneity for dichotomous outcomes by plotting the event rate in the intervention group relative to the event rate in the control group in each study. If the included studies are homogeneous, all points will be distributed in a straight line. If a study deviates too far from this line, it indicates significant heterogeneity in the study.

**Supplementary 8**

**PRISMA checklist**

| Section/topic | No. | Checklist item | Reported on page # |
| --- | --- | --- | --- |
| TITLE |  |  |  |
| Title | 1 | Identify the report as a systematic review, meta-analysis, or both. | 1 |
| ABSTRACT |  |  |  |
| Structured summary | 2 | Provide a structured summary including, as applicable: background; objectives; data sources; study eligibility criteria, participants, and interventions; study appraisal and synthesis methods; results; limitations; conclusions and implications of key findings;  systematic review registration number. | 1 |
| INTRODUCTION |  |  |  |
| Rationale | 3 | Describe the rationale for the review in the context of what is already known. | 2 |
| Objectives | 4 | Provide an explicit statement of questions being addressed with reference to participants,  interventions, comparisons, outcomes, and study design (PICOS). | 2 |
| METHODS |  |  |  |
| Protocol and registration | 5 | Indicate if a review protocol exists, if and where it can be accessed (e.g., Web address),  and, if available, provide registration information including registration number. | 2 |
| Eligibility criteria | 6 | Specify study characteristics (e.g., PICOS, length of follow-up) and report characteristics (e.g., years considered, language, publication status) used as criteria for eligibility, giving  rationale. | 3 |
| Information sources | 7 | Describe all information sources (e.g., databases with dates of coverage, contact with study  authors to identify additional studies) in the search and date last searched. | 3 |
| Search | 8 | Present full electronic search strategy for at least one database, including any limits used,  such that it could be repeated. | 3  Supplementary 2 |
| Study selection | 9 | State the process for selecting studies (i.e., screening, eligibility, included in systematic  review, and, if applicable, included in the meta-analysis). | 3 |
| Data collection process | 10 | Describe method of data extraction from reports (e.g., piloted forms, independently, in  duplicate) and any processes for obtaining and confirming data from investigators. | 3 |
| Data items | 11 | List and define all variables for which data were sought (e.g., PICOS, funding sources) and  any assumptions and simplifications made. | 3 |
| Risk of bias in individual studies | 12 | Describe methods used for assessing risk of bias of individual studies (including specifica- tion of whether this was done at the study or outcome level), and how this information is  to be used in any data synthesis. | 4 |
| Summary measures | 13 | State the principal summary measures (e.g., risk ratio, difference in means). | 4 |
| Synthesis of results | 14 | Describe the methods of handling data and combining results of studies, if done, including  measures of consistency (e.g., I2) for each meta-analysis. | 4 |
| Risk of bias across studies | 15 | Specify any assessment of risk of bias that may affect the cumulative evidence  (e.g., publication bias, selective reporting within studies). | 4 |
| Additional analyses | 16 | Describe methods of additional analyses (e.g., sensitivity or subgroup analyses,  meta-regression), if done, indicating which were pre-specified. | 4 |
| RESULTS |  |  |  |
| Study selection | 17 | Give numbers of studies screened, assessed for eligibility, and included in the review,  with reasons for exclusions at each stage, ideally with a flow diagram. | 4-5 |
| Study characteristics | 18 | For each study, present characteristics for which data were extracted  (e.g., study size, PICOS, follow-up period) and provide the citations. | 5 |
| Risk of bias within studies | 19 | Present data on risk of bias of each study and, if available, any outcome level assessment  (see item 12). | 5 |
| Results of individual studies | 20 | For all outcomes considered (benefits or harms), present, for each study:  (a) simple summary data for each intervention group (b) effect estimates and confidence intervals, ideally with a forest plot. | 5 |
| Synthesis of results | 21 | Present results of each meta-analysis done, including confidence intervals and measures  of consistency. | 5 |
| Risk of bias across studies | 22 | Present results of any assessment of risk of bias across studies (see Item 15). | 5 |
| Additional analysis | 23 | Give results of additional analyses, if done (e.g., sensitivity or subgroup analyses,  meta-regression [see Item 16]). | 5-7 |
| DISCUSSION |  |  |  |
| Summary of evidence | 24 | Summarize the main findings including the strength of evidence for each main outcome; consider their relevance to key groups (e.g., healthcare providers, users, and policy  makers). | 7 |
| Limitations | 25 | Discuss limitations at study and outcome level (e.g., risk of bias), and at review-level  (e.g., incomplete retrieval of identified research, reporting bias). | 9 |
| Conclusions | 26 | Provide a general interpretation of the results in the context of other evidence,  and implications for future research. | 9 |
| FUNDING |  |  |  |
| Funding | 27 | Describe sources of funding for the systematic review and other support  (e.g., supply of data); role of funders for the systematic review. | 9 |

**PRISMA, Preferred Reporting Items for Systematic reviews and Meta-Analyses**
